# Use of symptom-reporting tools to support endometriosis identification and management in primary care: a realist synthesis

**DOI:** 10.1136/bmjopen-2025-114764

**Published:** 2026-09-28

**Authors:** Mayara Silveira Bianchim, Rachel Joseph, Jacky Boivin, Jane Noyes

**Affiliations:** 1 School of Health Sciences, Bangor University, Bangor, UK; 2 School of Psychology and Women’s Health Research Wales Centre, Cardiff University, Cardiff, UK

**Keywords:** Primary Care, Chronic Pain, Community Participation, Decision Making

## Abstract

**Abstract:**

**Objectives:**

Endometriosis affects 5–10% of women of reproductive age and is associated with chronic pain, infertility and reduced quality of life. Symptom Reporting Tools (SRTs) may support identification and management in primary care, but evidence is limited. This review investigates what works, for whom, in what contexts and why, when using SRTs for endometriosis in primary care.

**Design:**

A realist synthesis was used to develop programme theories on how SRTs were intended to work for women and healthcare practitioners.

**Data sources:**

A purposive and iterative search was conducted across eight databases (Google Scholar, Press Reader, PubMed, Web of Science, LibrarySearch, APA PsycINFO via Ovid, Overton and Scopus) between January and April 2024 and updated in October 2025.

**Eligibility criteria:**

Studies were included if they focused on women, primary care professionals or SRT developers, and addressed symptom tracking, shared decision-making and diagnostic delays. Studies from low- and middle-income countries, non-English language or lacking SRT content were excluded.

**Data extraction and synthesis:**

Initial programme theories of how SRTs were intended to work for women and healthcare practitioners were developed, tested and refined with stakeholder and patient and public involvement and engagement.

**Results:**

This review included 102 studies: 83 informing initial theory development and 19 were used to refine these theories, covering a total of 22 266 participants aged between 13 and 70 years. Most studies included in the synthesis were cross-sectional (n=15). Evidence showed that independent SRT use could enhance symptom awareness, health literacy, self-management and quality of life. During appointments, SRTs might support communication and personalised treatment, potentially leading to more timely diagnosis and improved outcomes. However, without guidance, SRT use could also cause distress and disempowerment. Challenges included symptom variability and limited awareness of endometriosis and SRTs in primary care. Few studies explored the experiences of those unaware their symptoms could indicate endometriosis.

**Conclusion:**

Our findings highlight how and why using SRTs could support endometriosis identification and care in primary care. Evidence on their role in timely diagnosis and shared decision-making was limited. Implications for practice and further research were identified.

**PROSPERO registration number:**

CRD42024518266.

**STRENGTHS AND LIMITATIONS OF THIS STUDY**

- Our search was designed to include multiple evidence sources, including published studies, grey literature and social media.
- Stakeholder and patient and public involvement and engagement (PPIE) input was integrated throughout the synthesis.
- The realist approach enabled us to explore how and why symptom-reporting tools work across different contexts.
- Searches were restricted to English-language sources from high-income countries, which may limit transferability to other settings.
- Reliance on cross-sectional studies meant the synthesis mostly developed perceived, but not demonstrated, causal chains.

## Introduction

 Endometriosis is a disease difficult to diagnose and treat that affects approximately 5–10% of girls and women of reproductive age worldwide.^1–4^ It also affects people assigned female at birth, including transgender and non-binary people, as well as intersex and other gender-diverse people. Endometriosis is frequently associated with chronic pelvic pain, dysmenorrhea, infertility and poor quality of life.^5–7^ Despite its prevalence and significant impact,^4 8^ there is an average delay of 6–10 years in diagnosing the condition from the onset of symptoms.^9^ Heterogeneous presentations, the presence of non-specific symptoms (eg, fatigue) and unclear aetiology complicate diagnosis and management, with endometriosis often misdiagnosed as other conditions.^9^

Accurate clinical assessment is crucial for timely diagnosis and treatment.^10^ Symptom tracking can help with early identification, personalising treatment approaches and improving overall management and decision-making in primary care,^1 11^ National Institute for Health and Care Excellence (NICE)^12^ recommends that women with suspected endometriosis record their pain and symptoms and healthcare providers similarly advise symptom tracking to support diagnosis, guide evaluation and facilitate shared decision-making. Paper diaries and digital apps/tools (mobile or web) enable tracking of clinically relevant symptoms such as pain, mood and behaviour as they occur in daily life to reveal patterns and trends.^1^ We refer to these as Symptom Reporting Tools (SRTs). SRTs can help track changes in symptoms, identify symptom severity and when seeking medical help is warranted. Identification of symptoms that could be endometriosis and when to initiate the referral process are particularly important, given that timely referral from primary care to specialist care can expedite diagnosis and subsequent treatment of endometriosis.^13^

Several types of SRTs offer a platform to track endometriosis-related symptoms. This synthesis examined three types: (1) Generic period trackers which focus on menstrual cycle tracking and general gynaecological health, allowing users to log wide-ranging symptoms (eg, premenstrual). (2) Menstrual pain-specific SRTs, which focus on cycle-related pain (eg, cramps, back pain) that may indicate conditions like endometriosis. (3) Endometriosis-specific SRTs, designed for those with suspected or diagnosed endometriosis to track, report and manage symptoms. Most SRTs integrate health information on general well-being, menstruation, gynaecological health or specific conditions such as endometriosis.^1 2^

SRTs are relatively new and there is considerable variation in the use of these tools and their associated health information within primary care.^1 2^ Current research on the use of SRTs for endometriosis has focused on screening, diagnosis^1 2^ or their psychometric properties, such as validity and reliability.^3^ There is limited evidence on their role in supporting identification of possible endometriosis and management in primary care, and little is known about the various contexts and underlying causal processes that influence their utilisation and impact.

### Objectives and focus of review

#### Aims

The aim was to explore the use of publicly available SRTs to support endometriosis identification and management in primary care. The SPICE (Setting, Population/Perspective, Intervention/Interest, Comparison, Evaluation) framework (see online supplemental table S1)^14^ was used to formulate our review question: What works about SRTs used for endometriosis in primary care settings, for whom, in what contexts and why?

#### Objectives

1. Investigate whether SRTs track symptoms that are important to people with endometriosis.
2. Develop an overall understanding of how SRTs for endometriosis work in primary care.
3. Evaluate whether the use of SRTs for endometriosis in primary care resulted (or was perceived to result) in improved care (eg, better shared decision-making, timely diagnosis, facilitation of referrals).
4. Further develop and clarify the factors that create barriers and facilitators to optimal use of SRTs for endometriosis in primary care.
5. Make considerations for future use of SRT for endometriosis in primary care.

## Methods

We conducted a mixed-methods realist synthesis^15 16^ adhering to RAMESES (Realist And Meta-narrative Evidence Syntheses: Evolving Standards; online supplemental table S2)^17^ and the Preferred Reporting Items for Systematic Reviews and Meta-Analyses (PRISMA).^18 19^ The review protocol was registered with PROSPERO (CRD42024518266).

The realist approach to synthesis is an ideal method for exploring the use of SRTs in endometriosis care, as it provides a clear explanation of what works, for whom, how and why context influences outcomes. By focusing on perceived causal explanations and processes, realist synthesis generates findings that can guide developers, clinicians and policymakers in adapting and implementing SRTs effectively across diverse populations and settings.^15^ These explanations are formulated as initial programme theories (IPTs) that are then tested using a range of data sources in a focused synthesis. Within a realist synthesis, outcomes are understood to result from causal processes triggered by contextual factors, conceptualised as context–mechanism–outcome (CMO) configurations. The steps of the review were iterative and further detail about the method is provided in online supplemental material S1^16^.

### Stakeholder and patient and public involvement and engagement group

A patient and public involvement and engagement (PPIE) group including women with suspected (n=1) and confirmed diagnosis of endometriosis (n=10), general practitioner (GP) (n=1) and endometriosis nurse specialists (n=2) were involved from the initial funding proposal to the synthesis stage. PPIE was included during analysis and synthesis, to address evidence gaps and aid interpretation of findings. They identified barriers and facilitators to using SRTs in endometriosis care and provided suggestions for adapting identified SRTs to better meet user needs. Their input was central to shaping the final considerations for SRT use in clinical practice (for further see online supplemental material S2).

#### Initial programme theory

IPTs were developed through a combination of scoping the literature, searching websites, grey literature and social media, and discussions with team members with expert knowledge, as well as PPIE. IPTs were developed for the use of SRTs in diagnosing and managing endometriosis at various stages (unaware, suspected, confirmed) from the perspective of both women and healthcare professionals. By unaware, we refer to those who may be unaware that the symptoms they are experiencing could be endometriosis. Scoping the literature allowed us to understand what types of SRTs are commonly used for tracking symptoms of (suspected) endometriosis, as well as how these different SRTs are intended to work, to begin developing our IPTs.

##### Selection of SRTs

Selection of SRTs for in-depth examination was purposive, drawing on team expertise, literature searches, social media, patient blogs, and App Store searches. Inclusion criteria ensured:

- Focus on symptom tracking (eg, menstrual symptoms, severe pain, endometriosis).
- Broad range of formats (web apps, mobile apps, printable tools).
- Representation of diverse perspectives (charities, commercial companies, patient advocates, research bodies).
- Coverage of different aims but shared overarching purpose (supporting identification and reporting of symptoms possibly indicative of endometriosis).
- Relevance to the UK context, particularly Wales, or comparable settings.

A list of all the tools considered in the wider literature for inclusion is available in online supplemental table S3. Logic models were then created for a selected range of SRTs representing diverse perspectives and needs (eg, charities, advocates, research, commercial). Further details of these logic models are in (online supplemental figure S1a-g). Individual SRT logic models were synthesised into an overarching logic model, integrating insights from stakeholders and initial scoping to constitute the IPTs (see online supplemental figure S2; further details in online supplemental table S4). The IPTs assumed a linear process: If women use SRTs, they gain better awareness, communicate more effectively and receive better care.

### Searching and screening

Searches were purposive, transparent and iterative, and were conducted by two review authors (RJ and MSB) between January and April 2024 and updated in October 2025. All disagreements were resolved by discussion with a third author (JN and JB) involved if required. Search strategies were iterative and revisited throughout the synthesis to integrate emerging concepts and test developing programme theories.^16 17 19^

The primary search used Google Scholar and was supplemented by Press Reader, PubMed, Web of Science, LibrarySearch, APA PsycINFO (Ovid), and Scopus. Grey literature was retrieved via search engines, Overton and from stakeholder recommendations, and included policy documents, guidelines, blogs and news media. Search strings combined terms for endometriosis, SRTs, timely diagnosis and shared decision-making. Additional searches used berry-picking and CLUSTER (Citations, Lead authors, Unpublished materials, Scholar searches, Theories, Early examples and Related projects) techniques, and including snowballing, citation chasing and identification of related or ‘sibling’ studies.^20 21^ Searches were restricted to sources published from 2013 onwards, reflecting the period in which consumer SRTs became more widely available (see online supplemental table S5 for more information).

Titles and abstracts were screened independently by two authors (MSB and RJ) against predefined eligibility criteria (table 1) and relevance to theory development (table 2).^15^ Any disagreements were resolved through discussion with a third author when necessary (JN and JB). Included studies were assessed for rigour in terms of their contribution to theory testing and refinement. No studies were excluded solely on methodological quality, as partial elements could still yield valuable insights.^15 22^

**Table 1:** Inclusion and exclusion criteria

Inclusion criteria	Exclusion criteria
All study types (ie, quantitative, qualitative or mixed methods), documents, grey literature, online blogs, social media that:	
Studies/reports offering the perspective of women, girls and those assigned female at birth with suspected or diagnosed endometriosis, or symptoms that could be endometriosis on symptom-reporting, shared decision-making, diagnostic delay or timely diagnosis and the use of SRTs. Studies/reports offering the perspective of primary care professionals, endometriosis nurses and developers of SRTs on the tools in general, their use, shared decision-making, diagnostic delay and timely diagnosis. Studies/reports addressing endometriosis (or severe pelvic pain that could be endometriosis) that also mention SRTs, primary care, community, domestic, education or online settings. We also considered sources that did not explicitly mention primary care but indicated that the patient was at the beginning of their care journey or had not received referrals from primary care.	Studies/reports focused on low-income and middle-income healthcare settings. Non-English language papers. Studies/reports focused on discussing endometriosis or relevant websites, with no or little mention of SRTs.

SRT, Symptom Reporting Tool.

**Table 2:** Criteria used to determine relevance to theory development. Method adapted from^5 56^

Relevance to theory development	Any study, document type, source of information:
High relevance	Related to selected symptom reporting tools to be studied involving any of the criteria below or otherwise
	Offers the perspective of GP on patient use of symptom reporting tools for suspected or diagnosed endometriosis (Symptom Reporting Tools).
	Offers the perspective of patients on the use of Symptom Reporting Tools within primary care, in accessing care and obtaining diagnosis.
	Offers the perspective of endometriosis nurses/consultants or tool developers on the use of Symptom Reporting Tools in primary care or early in diagnostic journey.
	Observes or discusses quality of Symptom Reporting Tools and why some patients may or may not use them, or why healthcare providers may credit or discredit them.
	Relates to the communication barriers and facilitators between patients with diagnosed or suspected endometriosis and their GPs (relating to obtaining diagnosis/shared decision-making) and/or implementing advice at prediagnosis stage, and references Symptom Reporting Tools in some capacity.
	Describes and evidences why Symptom Reporting Tools impact care positively or negatively.
Medium relevance	Discusses ‘healthcare providers’ but does not specify if this is in primary care or otherwise, but does relate to early help-seeking for symptoms and obtaining endometriosis diagnosis and/or improving shared decision-making.
	Describes and provides evidence for why Symptom Reporting Tools generally, or in another related area of health, impact care positively or negatively.
	Discusses advances in fem-tech (period trackers, etc) in relation to endometriosis, severe period pain and potential impact on care.
Low relevance	Discusses Symptom Reporting Tools in relation to determining severity of endometriosis and/or placement of lesions and focuses little on the qualitative experiences of using such a tool in relation to diagnosis or shared decision-making.
	Focuses on artificial intelligence and its use in Symptom Reporting Tools.
	Focusing on aspects of menstrual cycle tracking with little focus on the use of the tool for endometriosis or qualitative reports of using the tool (for diagnosis and shared decision-making).
No relevance	Does not relate to any of the above.

GP, general practitioner.

At this stage, included full texts were organised into two categories depending on their relevance to theory development: (1) records used to inform theory development; and (2) records included in the synthesis.

### Realist synthesis

Data were extracted and managed by MSB and RJ using NVivo (V.20) and Excel, applying a coding framework developed from the IPTs.^15^ Study characteristics (ie, aims, design, participants, sample size, psychometric properties, sensitivity/specificity and engagement frequency) were recorded in Excel, while outcome data, relevance to IPTs and emerging CMO chains were coded in NVivo.^15 16^

Synthesis followed a retroductive and abductive process consistent with realist methodology, iteratively refining IPTs.^15 16^ Qualitative evidence (eg, participant quotes, thematic findings) was thematically coded against the IPT framework, while quantitative data (eg, engagement rates, acceptability, psychometric variables) were analysed descriptively in SPSS where possible. These datasets were integrated to test and elaborate CMO chains.

Analysis proceeded by comparing themes and quantitative evidence for each study to formulate chains of inference, linking contexts, mechanisms and outcomes.^23^ PPIE input was used throughout to address gaps, interpret findings and sense-check emerging CMOs. Factors influencing barriers and facilitators for the use of SRTs in informing endometriosis diagnosis and care were also identified. These, along with the programme theories and input from stakeholders and PPIE, then guided the development of practical considerations to enhance SRT utilisation in primary care.

The final narrative synthesis connected CMOs to explain how and why SRTs work, or fail, in different contexts, accounting for observed variability and identifying barriers, facilitators and implications for primary care.^15^ These findings, shaped by stakeholder and PPIE insights, informed the development of practical considerations for enhancing SRT use in clinical practice.

## Results

### Study selection, study and sample characteristics

After developing IPTs, 83 studies and reports informed theory development (see figure 1) and 19 studies were selected for the synthesis. These involved 22 266 participants (7384 participants were reported as female) aged 13–70 years. Most were conducted in the USA (n=8), UK (n=5), Australia (n=5) and New Zealand (n=4). Study designs were predominantly cross-sectional (n=15), with one longitudinal study, one case study, one pre–post single-group evaluation and one commentary. Eight studies used qualitative methods, six used quantitative methods and five adopted a mixed-methods approach (see online supplemental table S6). Five studies were excluded with reasons as described in online supplemental table S7.

**Figure 1:**
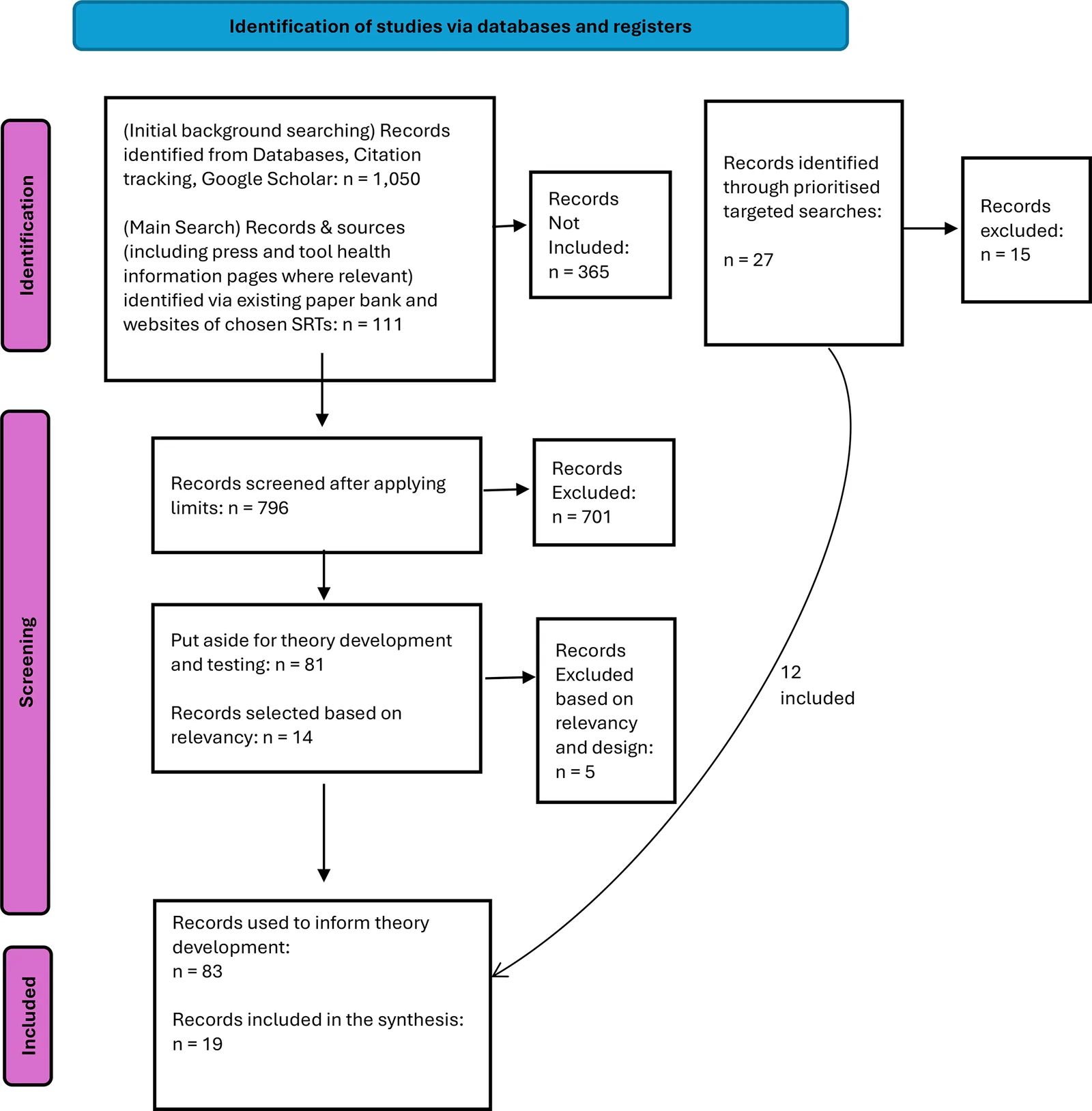
PRISMA flowchart outlining the processes of identification, screening and inclusion of studies. PRISMA, Preferred Reporting Items for Systematic Reviews and Meta-Analyses; SRTs, Symptom Reporting Tools.

SRTs included were: Flo (generic period tracker), Clue (generic period tracker), Period ImPact and Pain Assessment (PIPPA; menstrual pain specific), Raising Awareness Tool for Endometriosis (RATE; endometriosis specific), Phendo (endometriosis specific) (online supplemental table S8). One study investigated the use of an endometriosis-specific tracker developed by a Finnish patient organisation^24^ and six studies did not specify the SRTs used.^25–30^ Results on engagement, acceptability, sensitivity, specificity and reported effects of SRTs alongside study aims are described in online supplemental table S9 and were essential in developing the programme theories.

### Factors creating barriers and facilitators to SRT use

Online supplemental table S10 summarises seven key factors that generated 27 barriers and 21 facilitators for SRT use in primary care, informing refinement of the IPTs. These factors are related to tool design, functionality, accessibility, healthcare organisation, medical understanding, communication and societal influences. Barriers included exclusion of non-normative users (eg, irregular cycles), privacy concerns, high health literacy^31^ demands (eg, RATE), fragmented care, short GP appointments and menstrual stigma. Facilitators included personalisation, features like reminders, growing clinical awareness of endometriosis and educational resources for patients and GPs.

### Development of the final programme theories

Our analysis identified 16 CMOs explaining SRT use in endometriosis. Online supplemental table S11 presents the CMO chains and each study’s contribution to theory development, while figure 2 shows the updated logic model. The final programme theories suggest that SRTs may function differently when used independently versus within medical consultations and highlight additional mechanisms and potential negative effects, including system-level impacts. The synthesis narrative below provides a detailed explanation of the final programme theory, followed by sections describing the causal chains across different contexts.

**Figure 2:**
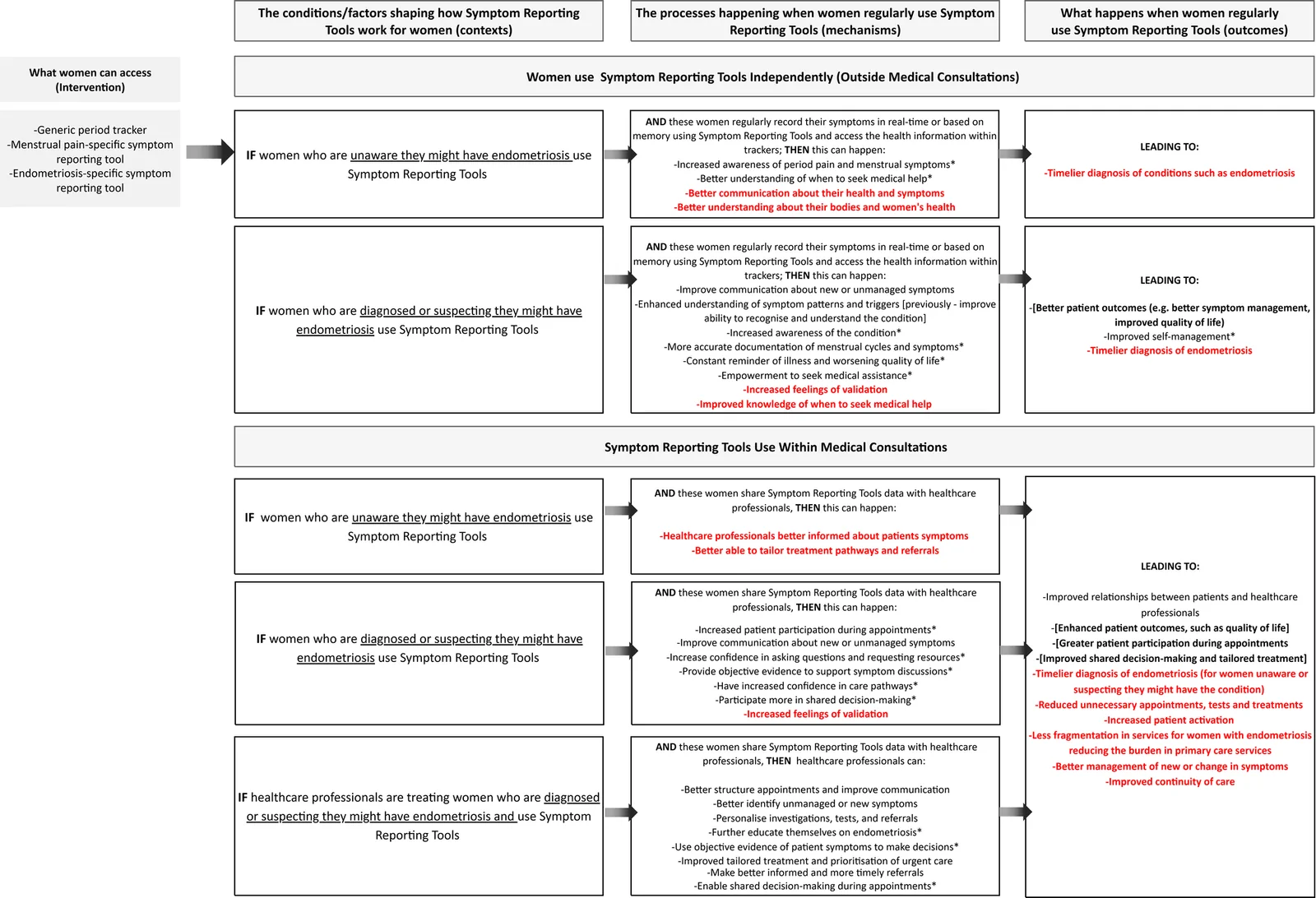
Logic model illustrating final programme theories from both women and healthcare professional perspectives across different contexts. Key: Black text shows IPTs that were tested and refined based on evidence. Bolded text indicates IPTs that could not be tested due to gaps in evidence but for which insights from patient and public involvement were informative. Text with asterisks * indicates new information could be added to IPTs after testing, refinement and further developing the IPT. Red text indicates a lack of evidence to test and refine the IPT. IPTs, initial programme theories.

### Women’s use of SRTs independently of medical encounters increased their awareness of symptoms and triggers, helping them achieve improved health literacy, better self-management and enhanced quality of life

The SRTs included in this realist synthesis were designed for independent use, helping women take control of their health. Women with endometriosis, whether suspected, diagnosed or unaware that their symptoms could be endometriosis, used these tools and associated resources to identify symptoms requiring medical attention, which could help them to have a better understanding of their symptoms and triggers and when to seek medical help. In women with diagnosed or suspected endometriosis, this could also lead to better health literacy and self-management, as well as enhanced quality of life. However, while SRTs provided significant benefits, they also had the potential to increase stress, sometimes worsening quality of life and increasing feelings of hopelessness.

#### Enhanced symptom awareness and medical help-seeking behaviour through SRTs

SRTs have the potential to help women (18–62 years) diagnosed with endometriosis better understand symptom patterns and triggers over time, independently of their healthcare providers.^24 32^ This was seen to increase awareness of the condition and contributing factors (eg, diet, weight, exercise, mood, sleep) associated with managing a chronic illness.^25 26 33^ SRTs appear to facilitate more accurate documentation of menstrual cycle and associated symptoms, including pain severity and timing,^25 34^ aiding memory recall and providing reliable data. However, when endometriosis is severe and symptoms are constant, it may be difficult to identify any pattern, leading to concerns about disease progression.^24^

Shea *et al*^32^ reported that women (regardless of diagnosis) were motivated to use generic SRTs for various reasons: to obtain cycle predictions (94%), identify symptom patterns (62%) or stay prepared (27%). The authors reported that tracking helped women understand their bodies and cycles (90%) and increased their perceived control over their health (72%). Women with suspected or diagnosed endometriosis who wanted to understand symptom progression demonstrated higher engagement with SRTs,^24 26 34^ which, in turn, led them to recognise consistent tracking as essential for identifying symptom patterns, changes and trends,^25 26 33–35^ as highlighted in the account below:

I just like looking at patterns and statistics…you need to have a data set to begin with to find out if something has actually changed. (woman, without a diagnosis of endometriosis, SRT used and age not specified)^30^

However, evidence on how and why these women used SRTs to begin with, as well as the mechanisms behind their increased awareness of symptoms and triggers, remains limited. In addition, various factors created barriers to consistent symptom tracking (online supplemental table S10). For instance, despite the convenience of smartphone-based SRTs, which were described as convenient and ‘easy to track [symptoms] as it’s on my phone’*,*^34 36^ women expressed the desire for personalisation of SRTs to aid tracking (eg, location-specific SRTs).^34^

Generic period trackers (eg, Flo) and menstrual pain-specific tools often helped women determine when to seek medical help for symptoms such as severe pain, regardless of diagnosis status.^34 35 37 38^ Insights from our PPIE group confirmed that these types of SRTs were useful in helping women recognise and track symptoms, especially before diagnosis and during the initial stages of seeking medical help. However, many women also refrained from seeking medical help due to embarrassment or belief that their symptoms were normal.^35 37^ In addition, the expected treatment pathways were often perceived as fragmented and unreliable, which discouraged many from seeking medical assistance.^25^

#### SRT function and functionality: women want to track more than their physical symptoms

Women, suspecting or diagnosed with endometriosis, expressed a desire to track not only the frequency of symptoms, but also the broader impact on their lives and mental health.^25–27 36^ More than 70% of women using a generic SRT (Clue) reported emotional symptoms, along with body pain and fatigue, as their main symptoms of concern.^32^ Some women described^25 27 36^ feeling empowered by tracking their emotional experiences, noting positive effects on their relationships and self-perception:

… [a SRT] has really helped me as an endometriosis/pmdd [Premenstrual dysphoric disorder] sufferer … I know when to take care with my diet and self-care … I know when I am likely to be anxious or taking things personally. (woman, SRT used and age not specified)^36^

However, current SRTs often lack the functionality to track emotional experiences,^25 26 34^ which can often result in fragmented tracking across different platforms^26^ or complete disengagement (online supplemental table S10). For some, this could exacerbate feelings of hopelessness and lack of agency.^24–26^ The evidence suggests that giving women autonomy over what they track could enable women to predict and understand their symptoms, thus helping them feel more prepared and in control of their health and lives.^28 29 34 36 39^ Despite that, most generic period trackers fell short of adequately capturing the diverse symptoms experienced by women with menstrual health conditions, despite the perceived value of features related to pain tracking and period prediction.^25 26 34 36 39^ In addition, most women were not aware of endometriosis-specific trackers.^34^

#### SRT use impact on self-management and quality of life

SRTs could help women identify self-management strategies that work for them.^35 37 38^ Indeed, a longitudinal study^40^ showed that women who regularly used an SRT to monitor their health independently of diagnosis (ie, 12 weeks or more) were more likely to answer questions related to self-management (eg, diet, medication) than those who did not use SRTs regularly.

The most common self-management strategies reported by women using a generic SRT (Clue) were pain medications (63%) and SRT use (57%).^32^ Many of the SRTs provided access to resources that helped users recognise signs associated with endometriosis and other menstrual disorders.^35 36^ These resources supported women (independent of diagnosis) in expanding or adapting their self-management approaches, which could lead to a greater sense of control.^34 37 40^ For example, Armour *et al*^38^ demonstrated that using the PIPPA tool and its associated health information helped some women (n=36, 48%) reconsider what constituted a normal period, which also helped increase their use of self-management strategies from 65% (n=48) to 86% (n=64). Similarly, Zhaunova *et al*^35^ and Patel *et al*^36^ found that using SRTs improved user knowledge of the menstrual cycle, with 88.98% (n=1292) and 83.1% (n=271) showing improvements, respectively. As a result, women could plan their lives with greater ease around anticipated challenges and implement self-care strategies, such as dietary adjustments, which enhanced the quality of life for those diagnosed with endometriosis^26 28 32 34 36^:

It’s a survival guide so, too, so that we can go out and enjoy ourselves every once in a while.^26^ (woman with endometriosis, SRT used and age not specified)

Despite the benefits of SRTs, some drawbacks were also reported. Some women experienced stress when their periods deviated from app predictions or when they forgot to enter data, which they felt undermined the accuracy of their tracking.^24 29^ For others, tracking acted as a continual reminder of ill health, contributing to reduced quality of life and increased feelings of hopelessness.^25 26^ However, contrasting evidence^32^ reported that clue (generic period tracker) users (64%) felt they would experience more health-related stress without tracking, suggesting that, for some, SRTs may reduce rather than exacerbate stress.

### SRTs use during medical appointments: impact on communication, women’s involvement, timely diagnosis and quality of life

The use of SRTs to inform medical appointments was perceived to increase women’s involvement in consultations, independent of diagnosis, but particularly for those with suspected or diagnosed endometriosis. This, in turn, could facilitate shared decision-making and help inform referrals to secondary care, potentially leading to a more personalised treatment approach and timelier diagnosis. However, causal evidence supporting these outcomes remains limited.

#### SRT use during medical appointments: enhancing communication

Women and healthcare professionals often had different conceptualisations of the woman’s health and symptoms. Women typically focused on day-to-day symptoms and their impact, while healthcare professionals tended to view the patient’s health over longer periods.^25^ This difference affected communication, as illustrated by one healthcare professional:

It can be really hard to sort through, if they are like ‘on this hour I felt this way and that way’. Most of us think in terms of weeks instead of days. (healthcare professional, specialty not specified)^25^

SRTs were reported to improve confidence and communication during appointments by enabling women, who suspected, were diagnosed with or were unaware they might have endometriosis, to document symptoms, identify symptom patterns and use correct terminology during appointments.^25 26 28 29 32 34 35 37 41^ Healthcare professionals also found SRTs valuable for structuring their communication, using SRT data to ask more relevant questions related to menstrual and gynaecological health.^24 25 41^ Some healthcare professionals acknowledged that women appreciated being asked about their symptoms,^25 41^ which created a sense of trust and strengthened the patient-provider relationship:

…a trust builder between [the patient] and I, so she can choose to show me this or not show me this…It’s a way for people that have been so minimized to say, ‘my pain is very real. (healthcare professional, specialty not specified)^25^

However, the perceived value and role of SRTs within medical appointments appeared to vary depending on clinicians’ focus and specialty. For example, endometriosis nurses viewed symptom tracking as beneficial for symptom awareness, whereas clinicians in other specialties, such as gynaecology, expressed concern that self-tracking might heighten women’s focus on their symptoms. As one professional remarked:

Sometimes I think it becomes never-ending, they monitor the body too much, every instance of pain, and then carefully report that. My work is to reduce some of that, not to self-track all the time because it makes self-tracking the focus*.* (sexual counsellor specializing in gynaecological conditions)^24^

#### SRT use during medical appointments: providing objective evidence of symptoms

SRTs were seen to provide objective evidence of women’s symptoms, which helped structure appointments and prompt GPs to ask more pertinent questions, improving recognition of problematic menstrual pain and other symptoms.^28 29 32 34 41^ Some women used SRT data as evidence of their symptoms during consultations^37 41^:

Having used a tracking app for years, I could rely on objective data to convince doctors that I need surgery to confirm and remove endometriosis. (survey Respondent, generic SRT, age not specified)^34^

However, insights from PPIE revealed that it could be challenging for women to use SRTs as evidence of their symptoms even when they had a diagnosis of endometriosis:

And my GP was [initially] very good at looking at my tracker (…) but since I’ve been diagnosed my GP that I’ve got now doesn’t help me with endometriosis at all. They just go. Have you phoned an endometriosis specialist and they will instantly refer me back to the specialist. (PPIE group)

The group also highlighted that, following diagnosis, the emergence of new symptoms (likely attributable to endometriosis) could create a sense of returning to the starting point in their endometriosis care (eg, ‘back at square one’, PPIE group). This issue is further complicated by the wide range of SRTs, each using different tracking metrics and data formats. This variation can make it challenging for healthcare professionals to interpret the data within the limited time available during appointments.^25^ Women (particularly those diagnosed with endometriosis) often felt dismissed or distressed when doctors did not value or interpret their SRT information, or when there was insufficient time to look through it.^25 26 32^

Women (diagnosed with endometriosis) also faced challenges when deciding which symptoms to track and what information to prioritise during their limited appointment time.^25^ The continuous nature of the condition made it difficult to pinpoint symptom onset, duration and patterns (eg, frequency), and which symptom to track.^25^ Which is further exacerbated by certain SRTs providing numerous tracking options.^25 26 34^ During the synthesis process, we hypothesised that increasing women’s health literacy can aid women in identifying which symptoms to track and which require further investigation.^35 37^ GPs could also play a crucial role by advising women on which symptoms to track (when choice exists), focusing on those most relevant for informing clinical decisions.^25 41^

#### Empowering women: enhanced knowledge and self-reflection through SRTs increase active engagement in medical appointments

Frequent SRTs use, both endometriosis-specific or generic period trackers, was seen to empower women to become more actively involved during medical appointments.^35 41^ For instance, women (independently of diagnosis) who frequently used the generic period tracker Flo or Clue (several times a day to several times a week) were able to recognise concerns related to endometriosis and felt more confident in asking their doctors questions about their health.^25 32 35^ This increased involvement extended to women with lower levels of education, who reported feeling more confident in requesting necessary resources from healthcare providers.^35^

However, increased patient involvement could sometimes strain the patient-professional relationship by challenging traditional power dynamics.^25 26^ Furthermore, stress was seen to arise for both healthcare professionals and women when misinformation from unreliable sources had to be corrected by professionals.^25^ In addition, higher patient involvement during consultations, without effective collaboration with healthcare providers, often led to increased stress and negative perceptions of the healthcare experience. For example, participants in Pichon *et al*^25^ described feeling stressed when they were more involved in their care without proper guidance, resulting in feelings of blame when treatments were inadequate or unsuccessful. PPIE insights further highlighted the finding that acknowledging patients’ lived expertise without providing additional support could exacerbate these negative feelings^25^:

…If I was to get a pound for each time a doctor said, ‘look you know more than us’ – I even had it with my daughter, you know. They said, ‘well, look, you’ve got endometriosis [mother], you know way more than us so you’re probably better off explaining to her [daughter].’ And it’s like, whoa…that could be really unsettling for anyone who doesn’t actually know much about endometriosis. (PPIE group)

In this context, SRTs can help empower patients to engage in informed discussions with healthcare professionals, creating a more collaborative approach to decision-making.^25 41^

### Using SRTs informs the direction of care potentially leading to timelier diagnosis

Some GPs found that SRTs helped structure appointments, assisting them in identifying potential issues and discussing overlooked topics such as menstrual health.^32 41^ The use of SRTs was also reported to enhance GPs’ confidence in their clinical decisions, particularly when working with women not yet diagnosed with endometriosis.^41^ This approach could lead to more personalised treatment in primary care (eg, cognitive behavioural therapy, physiotherapy or prescribed medications), as well as streamlined tests and more efficient referrals to secondary and tertiary care.^41^ Frayne *et al*^41^ described that using the RATE tool to inform appointments prompted further investigations, regardless of an endometriosis diagnosis. Armour *et al*^38^ also found that 56% (n=42) of women using the PIPPA tool during GP appointments were referred to gynaecology. The use of Clue helped women identify health issues and, in some cases, reach a diagnosis:

I was misdiagnosed with IBS as a teenager and only through tracking my cycle with Clue over the past year did I realise it was likely a gynaecological issue… I was diagnosed with stage IV endometriosis. (survey respondent, generic SRT, age not specified)^32^

However, some GPs acknowledged that while beneficial, SRTs required high levels of health literacy from their users.^41^ This challenge is compounded by the considerable variation in the quality and content of SRTs.^42–48^ Nevertheless, during the synthesis process, we hypothesise that SRT use and associated resources can improve health literacy, empowering women to seek medical assistance while increasing their trust in the direction of their care.

## Discussion

To our knowledge, this is the first realist synthesis to examine the use of SRTs in supporting the identification and management of endometriosis in primary care. The findings offer new insights into their use both independently and during medical consultations, alongside practical considerations (table 3).

**Table 3:** Considerations for the implementation and use of SRTs as part of endometriosis identification and management

Main considerations	Description
Further research and resources	More research including patient and public involvement (patients, third-sector organisations and healthcare professionals) is needed to investigate what works about SRTs, for whom, how and why for symptoms of suspected and confirmed endometriosis. More resources are needed to support and guide people in finding and using SRTs that are most suitable for their current health needs (ie, for suspected endometriosis). SRTs should be used in combination with other approaches as part of a holistic strategy. More research is needed to investigate how SRTs are used for different reasons (ie, for monitoring, self-management or/and endometriosis identification) and how this can impact tool use and its effects. More research is needed on SRTs to support initiatives to improve menstrual health education and awareness of endometriosis, as well as care. More research is needed specifically on patient outcomes from SRT use in endometriosis (eg, timely diagnosis, shared decision-making and shared goals), and whether these outcomes vary depending on what SRT is used. More research is needed on how baseline psychological factors (eg, anxiety, symptom hypervigilance) may modify the use and impact of SRTs in endometriosis. More research is needed on whether continuous use of SRTs can increase stress and anxiety.
Awareness of the different types of SRTs (eg, endometriosis specific trackers vs period pain and menstrual trackers)	Resources and training should be co-produced with a wide range of relevant stakeholders and focus on increasing awareness of evidence-based menstrual and endometriosis-specific SRTs to the public and healthcare professionals. Resources and training signposting to SRTs and their use should be disseminated via organisations (education and health settings). For education settings, awareness of SRTs and the dissemination of information could happen via lessons, assemblies and external speakers, as well as via email or letters. For workplace settings, dissemination could happen via online systems, internal support groups, meetings regarding health and well-being. Information within healthcare settings may be disseminated via leaflets and electronic notices for patients. For healthcare professionals, particularly primary care, we suggest that information and awareness of SRTs for menstrual disturbance and/or symptoms of endometriosis is delivered via training. For patients, information may be disseminated on primary care services (eg, notice boards, screens and via healthcare professionals). Social media (eg, Facebook, TikTok) can also help to increase awareness among the general population.
Resources to inform the use of SRTs	Resources are needed to provide tailored suggestions on how and what to track when a variety of symptoms are suggested in generic SRTs. Suggestions could also include how to track symptoms in a way that may be helpful for healthcare providers, depending on diagnostic status. Healthcare providers could help women select what to track (independently or together) and use these to inform treatment strategies and diagnosis.
Embedding SRTs in guidelines and trusted websites to support endometriosis management and care	Evidence-based SRTs should be embedded within endometriosis guidelines, such as NICE or ESHRE guidelines. Furthermore, information about SRTs should also be included in primary care platforms such as Health Pathways (https://healthpathwaysglobal.org/), relevant third-sector organisation websites and also health websites.
Enabling personalisation of tools	SRTs should enable personalisation and allow users to track their physical and emotional experiences and the impact of symptoms on their lives. SRTs should also enable users to track the mental health impact on their lives, particularly for those using the tracker for the purposes of monitoring and self-management. Further attention and consideration are warranted regarding tool design, associated health information and functionality. Particularly, regarding period predictions, (less))gendered designs and assumptions, as well as location (eg, suited to women within a specific country), life stages and specific health goals. To aid healthcare practitioners in reviewing the SRTs, the presentation of SRTs data (ie, symptom reports or graphs) should be standardised across tools. This could save time during appointments as there would be less time spent trying to interpret data presented in a variety of different ways.
Considerations for clinical practice	Advice and guidance from tertiary care may help healthcare professionals working in primary care and their patients navigate the uncertainty of endometriosis and the difficulty in choosing the correct care pathway. SRTs should not be used to dismiss patients’ concerns or replace a clinical investigation, but rather, they should be used as part of a holistic approach to care. SRTs can help primary care clinicians better structure the appointment and ask more relevant questions related to menstrual and gynaecological health. Clinicians can advise women on what symptoms to track to help inform clinical decisions
Privacy concerns and confidentiality	Generic and specific SRTs should protect users’ data and confidentiality. Women are more likely to engage and adhere to SRTs they perceive as safe (eg, app developed or backed by a governmental body).

ESHRE, European Society of Human Reproduction and Embryology; NICE, National Institute for Health and Care Excellence; SRTs, Symptom Reporting Tools.

### Strengths and limitations

This study will inform a large-scale realist evaluation which will focus on the use of the Endometriosis Cymru Symptom Tracking and Reporting Tool (ESTR; https://endometriosis.cymru/srt/) in Wales. It is worth noting that some authors of this study were involved in developing ESTR. Additionally, by beginning to unpack how and why SRTs may support endometriosis symptom identification and management in primary care across different contexts, we can start to ensure more meaningful use of these tools, both in practice and at the individual level, to potentially facilitate timelier diagnosis.

Several limitations warrant consideration. The study focused exclusively on English-language sources from high-income countries, limiting the applicability and transferability of the findings to other contexts. Additionally, most included studies were cross-sectional, which means findings represent participants’ perceived causal chains, and not demonstrated causal chains. Therefore, longitudinal and prospective research will be needed before specific clinical recommendations can be made. Studies were appraised for relevance and rigour based on team and stakeholder consensus, rather than a validated instrument. While consistent with realist methodology, this relies on interpretative judgement and may limit reproducibility. Preprints were included to capture the latest evidence available, in line with realist methodology, and their status was flagged in online supplemental table S6. However, as preprint findings can be revised at peer review, this represents a limitation of the evidence included. Included studies did not specify how or whether SRTs work for non-cisgender participants, leaving a gap in our understanding of SRTs’ use for these populations. Despite these limitations, the synthesis encompassed evidence from multiple sources, including grey literature and social media and focused on specific tools commonly used in the UK, and other English-speaking countries. Insights from PPIE were integrated throughout the study, increasing the relevance of findings.

### Comparing and contrasting with broader literature

We found that SRTs work effectively when there is consistent independent use and sufficient health literacy. As a result, SRTs can help increase awareness of symptom patterns and prompt women to seek medical help, thereby improving self-management and quality of life. This aligns with evidence from outside the specific context of SRT use, which highlights the importance of understanding endometriosis and its symptoms to enable effective self-management.^49^ Roomaney and Kagee^50^ similarly suggested that a comprehensive understanding of the impact of endometriosis on life can facilitate the acceptance of non-medical treatments (where relevant), supporting the role of SRTs in shared decision-making about treatment options.^49^

Effective communication and collaboration between patients and healthcare professionals are essential for managing endometriosis in primary care, facilitating specialist referrals and enabling early diagnosis.^51^ However, evidence highlights ongoing discrepancies in how patients and professionals communicate about symptoms, particularly pain.^52^ SRTs have the potential to bridge this gap by enhancing communication, increasing women’s involvement in consultations and contributing to more personalised treatment and referral decisions. For this to happen, clinicians need to be receptive and open to discuss SRT data during appointments.

While SRTs hold potential, their use in medical consultations is not yet optimised. A key issue is that SRTs often fail to capture the lived experiences and emotional impact of symptoms, which women with suspected or confirmed endometriosis reported as important to share in consultations. Additionally, when SRTs do not perform as expected (eg, SRTs fail to capture the lived emotional impact of symptoms, and when clinicians do not engage with SRT data), they can lead to confusion or miscommunication between patients and healthcare professionals. This is particularly problematic, as healthcare professionals may consequently unintentionally normalise severe period pain and endometriosis symptoms, leaving women feeling dismissed and unheard.^53 54^ Our findings suggested that when SRTs are effective, they can help women communicate their symptoms more clearly, providing objective evidence that counters this normalisation. This can lead to more meaningful discussions during consultations of clinically relevant symptoms, potentially resulting in improved treatment outcomes and earlier referral or diagnosis.

It is important to note that our focus on primary care reflects the situation in the UK, where patients typically access healthcare through GPs before being referred to specialists (eg, gynaecologist). In healthcare systems where direct self-referral to gynaecology is possible, there may be different considerations, potentially affecting how and when SRTs are used. Note, however, that many included studies were implemented in countries where direct routes to specialists are possible. Further, evidence investigating how and why individuals use the different types of SRTs (ie, generic, pain-specific or endometriosis-specific) in primary care for endometriosis symptoms (diagnosed or not), and whether their use leads to more timely diagnosis and improved patient outcomes, is currently limited.

There is also a lack of research exploring how various contextual factors (eg, gender identity, disability, ethnicity, sexual identity and socioeconomic status) influence the effectiveness and accessibility of these tools. For instance, while many of the studies seem to have included women who were unaware that their symptoms might indicate endometriosis, these studies did not investigate this group in isolation. In addition, there was no evidence comparing the effects of monthly versus daily symptom tracking. Moreover, few studies have examined healthcare professionals’ perspectives on SRTs during medical encounters.^25 34 40 41 55^ This gap in evidence has hindered our ability to draw firm conclusions about how SRTs contribute to better diagnosis and care for women, indicating the need for future research to explore their impact across diverse populations and healthcare settings.

More empirical evidence is needed to demonstrate whether and how SRTs can provide value in primary care to help inform the use, design and implementation of SRTs in these settings. Future research should also examine the proposed causal mechanisms by which SRTs operate in primary care. When developing and implementing SRTs and associated resources, future studies could ensure the accessibility and effectiveness of SRTs by involving multiple stakeholders, including patients, healthcare practitioners and service providers. Additionally, where appropriate, future research should address whether the impact of the digital divide is significant when implementing SRTs in primary care.

### Considerations for implementation

In table 3, we present considerations for the implementation of SRTs where relevant. These were informed by data from the included studies (ie, 83 studies for theory development and 19 for the synthesis) as well as insights from PPIE and stakeholders (eg, healthcare professionals and those with lived experience of endometriosis). While the focus was mainly on primary care, we see no reason why these would not also contribute to implementation in secondary care. As shown in table 3, implementation could be optimised with implementation research but also with a better understanding of what works for different users and in which contexts. SRTs should be part of a holistic strategy wherever implemented, and more awareness of the different types of SRTs available is needed among patients and healthcare professionals. Dissemination of SRT-related information should take place in educational, workplace and healthcare settings, as well as through social media, and as relevant, to target different groups. Resources should guide users on how to track symptoms effectively, and healthcare providers could support this process to improve communication about symptoms and shared decision-making about diagnosis and treatment. In considering healthcare providers, standardising the presentation of SRT data would help healthcare professionals’ interpretation. Ensuring privacy protection and embedding SRTs into trusted guidelines could facilitate their use. Finally, SRTs should not replace clinical investigations but should be used to complement a broader care strategy.

## Conclusions

SRTs appear most useful for women who actively seek them out, use them consistently and feel confident sharing their records with healthcare professionals. When professionals are receptive and value the information, SRTs can help structure appointments and improve communication. However, concerns remain about the risk of misinformation from unreliable sources. Better integration of SRTs into routine care pathways could enhance diagnosis timelines, support shared decision-making and improve access to and monitoring of treatment. Further theory work leading to an overarching explanatory framework for symptom-reporting, help-seeking and self-management in health is needed.

## Supplementary material

10.1136/bmjopen-2025-114764 online supplemental file 1

## Data Availability

All data relevant to the study are included in the article or uploaded as supplementary information.

## References

[1] Sivajohan B, Elgendi M, Menon C (2022). Clinical use of artificial intelligence in endometriosis: a scoping review. *NPJ Digit Med*.

[2] Surrey E, Carter CM, Soliman AM (2017). Patient-completed or symptom-based screening tools for endometriosis: a scoping review. Arch Gynecol Obstet.

[3] Parker MA, Kent AL, Sneddon A (2022). The Menstrual Disorder of Teenagers (MDOT) Study No. 2: Period ImPact and Pain Assessment (PIPPA) Tool Validation in a Large Population-Based Cross-Sectional Study of Australian Teenagers. J Pediatr Adolesc Gynecol.

[4] Taylor HS, Kotlyar AM, Flores VA (2021). Endometriosis is a chronic systemic disease: clinical challenges and novel innovations. Lancet.

[5] Simoens S, Dunselman G, Dirksen C (2012). The burden of endometriosis: costs and quality of life of women with endometriosis and treated in referral centres. Hum Reprod.

[6] Pontoppidan K, Olovsson M, Grundström H (2023). Clinical factors associated with quality of life among women with endometriosis: a cross-sectional study. BMC Womens Health.

[7] Cox H, Henderson L, Wood R (2003). Learning to take charge: women’s experiences of living with endometriosis. Complement Ther Nurs Midwifery.

[8] Nnoaham KE, Hummelshoj L, Webster P (2011). Impact of endometriosis on quality of life and work productivity: a multicenter study across ten countries. Fertil Steril.

[9] De Corte P, Klinghardt M, von Stockum S (2025). Time to Diagnose Endometriosis: Current Status, Challenges and Regional Characteristics-A Systematic Literature Review. BJOG.

[10] Allaire C, Bedaiwy MA, Yong PJ (2023). Diagnosis and management of endometriosis. CMAJ.

[11] Edgley K, Horne AW, Saunders PTK (2023). Symptom tracking in endometriosis using digital technologies: Knowns, unknowns, and future prospects. Cell Rep Med.

[12] National Institute for Health and Care Excellence (2024). Endometriosis: diagnosis and management. NICE guideline [NG73].

[13] Pickett C, Foster WG, Agarwal S (2023). Current endometriosis care and opportunities for improvement. Reprod Fertil.

[14] Harris JL, Booth A, Cargo M (2018). Cochrane Qualitative and Implementation Methods Group guidance series-paper 2: methods for question formulation, searching, and protocol development for qualitative evidence synthesis. J Clin Epidemiol.

[15] Pawson R, Greenhalgh T, Harvey G (2005). Realist review--a new method of systematic review designed for complex policy interventions. J Health Serv Res Policy.

[16] Rycroft-Malone J, McCormack B, Hutchinson AM (2012). Realist synthesis: illustrating the method for implementation research. Implement Sci.

[17] Wong G, Greenhalgh T, Westhorp G (2013). RAMESES publication standards: realist syntheses. BMC Med.

[18] Page MJ, McKenzie JE, Bossuyt PM (2021). The PRISMA 2020 statement: an updated guideline for reporting systematic reviews. BMJ.

[19] Page MJ, Moher D, Bossuyt PM (2021). PRISMA 2020 explanation and elaboration: updated guidance and exemplars for reporting systematic reviews. BMJ.

[20] Tsang A, Maden M (2021). CLUSTER searching approach to inform evidence syntheses: A methodological review. Res Synth Methods.

[21] Booth A, Briscoe S, Wright JM (2020). The “realist search”: A systematic scoping review of current practice and reporting. Res Synth Methods.

[22] Kastner M, Estey E, Perrier L (2011). Understanding the relationship between the perceived characteristics of clinical practice guidelines and their uptake: protocol for a realist review. Implement Sci.

[23] Thomas J, Harden A (2008). Methods for the thematic synthesis of qualitative research in systematic reviews. BMC Med Res Methodol.

[24] Oikkonen V, Temmes M (2025). Self-tracking in endometriosis: Evolving expectations around a gynecological app developed by a Finnish patient organization. Soc Stud Sci.

[25] Pichon A, Schiffer K, Horan E (2021). Divided We Stand: The Collaborative Work of Patients and Providers in an Enigmatic Chronic Disease. *Proc ACM Hum Comput Interact*.

[26] McKillop M, Voigt N, Schnall R (2016). Exploring self-tracking as a participatory research activity among women with endometriosis. J Particip Med.

[27] Ford A, De Togni G, Miller L (2021). Hormonal Health: Period Tracking Apps, Wellness, and Self-Management in the Era of Surveillance Capitalism. *Engag Sci Technol Soc*.

[28] Levy J, Romo-Avilés N (2019). “A good little tool to get to know yourself a bit better”: a qualitative study on users’ experiences of app-supported menstrual tracking in Europe. BMC Public Health.

[29] Broad A, Biswakarma R, Harper JC (2022). A survey of women’s experiences of using period tracker applications: Attitudes, ovulation prediction and how the accuracy of the app in predicting period start dates affects their feelings and behaviours. Womens Health (Lond).

[30] Hohmann-Marriott B (2023). Periods as powerful data: user understandings of menstrual app data and information. New Media Soc.

[31] Nutbeam D (1998). Health promotion glossary. Health Promot Int.

[32] Shea AA, Weber K, Hainer C Menstrual tracking app use in menstrual health self-management: a cross-sectional survey of user concerns, goals, and strategies. In Review.

[33] Hong M, Rajaguru V, Kim K (2024). Menstrual Cycle Management and Period Tracker App Use in Millennial and Generation Z Individuals: Mixed Methods Study. J Med Internet Res.

[34] Hohmann-Marriott BE, Williams TA, Girling JE (2023). The role of menstrual apps in healthcare: provider and patient perspectives. N Z Med J.

[35] Zhaunova L, Bamford R, Radovic T (2023). Characterization of Self-reported Improvements in Knowledge and Health Among Users of Flo Period Tracking App: Cross-sectional Survey. JMIR Mhealth Uhealth.

[36] Patel U, Broad A, Biswakarma R (2024). Experiences of users of period tracking apps: which app, frequency of use, data input and output and attitudes. Reprod Biomed Online.

[37] Armour M, Avery J, Leonardi M (2022). Lessons from implementing the Australian National Action Plan for Endometriosis. Reprod Fertil.

[38] Armour M, Parry K, Curry C (2022). Evaluation of a web-based resource to improve menstrual health literacy and self-management in young women. J Psychosom Res.

[39] Scheck SM, Hohmann-Marriott B, Faughnan G (2023). A survey exploring the use of symptom diary mobile apps for endometriosis. J Endometr Pelvic Pain Disord.

[40] Elhadad N, Urteaga I, Lipsky-Gorman S (2022). User engagement metrics and patterns in phendo, an endometriosis research mobile app. In Review.

[41] Frayne J, Milroy T, Rook C (2024). Acceptability of using the Raising Awareness Tool for Endometriosis (RATE) in general practice: a mixed methods pilot study. Aust N Z J Obstet Gynaecol.

[42] Epstein DA, Lee NB, Kang JH (2017). Examining menstrual tracking to inform the design of personal informatics tools. https://dl.acm.org/doi/proceedings/10.1145/3025453.

[43] Gkrozou F, Waters N (2019). A preliminary review of the mHealth apps focused in endometriosis and chronic pelvic pain. Health Sci J.

[44] Wong DC, Nwe K, Evans R (2021). Quantity and type of peer-reviewed evidence for popular free medical apps: Cross-sectional review. Int J Med Inform.

[45] Karasneh RA, Al-Azzam SI, Alzoubi KH (2020). Smartphone Applications for Period Tracking: Rating and Behavioral Change among Women Users. Obstet Gynecol Int.

[46] Moglia ML, Nguyen HV, Chyjek K (2016). Evaluation of Smartphone Menstrual Cycle Tracking Applications Using an Adapted APPLICATIONS Scoring System. Obstet Gynecol.

[47] Hirsch M, Aggarwal S, Barker C (2017). Googling endometriosis: a systematic review of information available on the Internet. Am J Obstet Gynecol.

[48] Dinh T, Flaxman T, Shea K (2022). What Are Patients Reading? Quality Assessment of Endometriosis Information on the Internet. J Obstet Gynaecol Can.

[49] Leonardi M, Horne AW, Vincent K (2020). Self-management strategies to consider to combat endometriosis symptoms during the COVID-19 pandemic. *Hum Reprod Open*.

[50] Roomaney R, Kagee A (2016). Coping strategies employed by women with endometriosis in a public health-care setting. J Health Psychol.

[51] Dixon S, McNiven A, Talbot A (2021). Navigating possible endometriosis in primary care: a qualitative study of GP perspectives. Br J Gen Pract.

[52] Bullo S, Weckesser A (2021). Addressing Challenges in Endometriosis Pain Communication Between Patients and Doctors: The Role of Language. *Front Glob Womens Health*.

[53] Requadt E, Nahlik AJ, Jacobsen A (2024). Patient experiences of endometriosis diagnosis: A mixed methods approach. BJOG.

[54] Young K, Fisher J, Kirkman M (2020). Partners instead of patients: women negotiating power and knowledge within medical encounters for endometriosis. Fem Psychol.

[55] Peven K, Wickham AP, Wilks O (2023). Assessment of a Digital Symptom Checker Tool’s Accuracy in Suggesting Reproductive Health Conditions: Clinical Vignettes Study. JMIR Mhealth Uhealth.

[56] Kantilal K, Hardeman W, Whiteside H (2020). Realist review protocol for understanding the real-world barriers and enablers to practitioners implementing self-management support to people living with and beyond cancer. BMJ Open.

