# Supplementary material for: Use of symptom-reporting tools to support endometriosis identification and management in primary care: a realist synthesis
Source: BMJ Open. 2026 Sep 28;16(9):e114764. doi: 10.1136/bmjopen-2025-114764 (PMC13629848; doi:10.1136/bmjopen-2025-114764)
Supplement: Supplementary file 1 [file bmjopen-2025-114764.reviewer_comments.pdf]

## PEER REVIEW HISTORY

BMJ Open publishes all reviews undertaken for accepted manuscripts. Reviewers are asked to complete a checklist review form and are provided with free text boxes to elaborate on their assessment. These free text comments are reproduced below.

### ARTICLE DETAILS

**Title (Provisional)**

The use of Symptom-Reporting Tools to support Endometriosis Identification and Management in Primary Care: A Realist Synthesis

**Authors**

Silveira Bianchim, Mayara; Joseph, Rachel; Boivin, Jacky; Noyes, Jane

---

### VERSION 1 - REVIEW

---

| | |
|---|---|
| **Reviewer** | **1** |
| **Name** | **Ban FrangeŽ, Helena** |
| **Affiliation** | **Division of Gynaecology and Obstetrics, University Medical Centre Ljubljana** |
| **Date** | **30-Jan-2026** |
| **COI** | **None** |

---

This manuscript presents a well-conducted realist synthesis exploring how symptom-reporting tools (SRTs) are used in the identification and management of endometriosis in primary care. The methodology is appropriate, transparently reported, and aligned with RAMESES and PRISMA guidance. The inclusion of stakeholder and PPIE input is a clear strength.

However, from a clinical perspective, the manuscript remains largely explanatory and does not sufficiently clarify the implications for routine practice. While both potential benefits and harms of SRT use are described, the synthesis does not provide a clear appraisal of when SRTs are likely to be helpful versus potentially detrimental.

In particular, the review does not adequately consider baseline psychological vulnerability, such as anxiety or symptom hypervigilance, which are common in women with endometriosis and may substantially modify the impact of symptom tracking. Although increased distress and stress are acknowledged as possible outcomes, these factors are not explicitly integrated into the programme theories or context–mechanism–outcome configurations.

As a result, the conclusions remain theoretical and difficult to translate into clinical decision-making. The manuscript would be strengthened by:

More explicit acknowledgement of the limits of clinical applicability.

Clearer differentiation between perceived benefits and demonstrated effects.

Integration of mental health factors (e.g. anxiety, distress) as key contextual modifiers.

A more explicit clinical “bottom line” to guide primary care interpretation.

Overall, this is a valuable and methodologically sound contribution, but greater clarity regarding its clinical relevance and boundaries would improve its usefulness for practitioners.

---

**Reviewer** **2**

**Name** **Doust, Jenny**

**Affiliation** **The University of Queensland Faculty of Medicine and Biomedical Sciences**

**Date** **29-Apr-2026**

**COI** **None**

---

The use of Symptom-Reporting Tools to support Endometriosis Identification and Management in Primary Care: A Realist Synthesis

The manuscript is a realist synthesis of the literature investigating the use of symptom-reporting tools by people who have, or suspect they might have, endometriosis, before or in conjunction with attendance at a primary care health service.

This is an ambitious and comprehensive paper. It fulfils a useful role to assist the understanding of how such tools might be better developed and used in primary care. The authors have also developed a new endometriosis symptom-reporting tool, which is included in the synthesis. The results of this synthesis will inform further evaluation of this new tool.

The authors provide a well-thought-out description of what a realist synthesis is and of the review's objectives. The extensive use of PPI in developing these objectives is evident in the themes' relevance to patients and clinical practice.

I have a few comments to improve the manuscript:

1) The research questions did not sufficiently differentiate between a symptom tracker used to identify whether a woman might have endometriosis and one used to monitor the condition and/or when there is a need for further treatment or referral. This distinction could be quite important in the tool's application. For example, mental health effects are clearly extremely important for understanding the impact of the condition on women’s

quality of life. However, mental health effects may or may not be helpful in identifying who is at the highest risk of having the condition. This is particularly important when it comes to the authors' suggestion that the tool be personalised. This could be important for monitoring the condition, but not for identifying it.

2) Symptom-reporting tools are quite burdensome for patients to complete and for clinicians to interpret during a consultation. It remains unclear to me whether a system in which a woman tracks daily symptoms is preferable to a monthly symptom checklist. The burden these tools place on both patients and clinicians should be considered among the potential harms. The burden of symptom tracking and the potential for it to be harmful for some patients are illustrated in some of the quotes in the synthesis, but this needs to be highlighted more in the abstract and conclusion.

3) It was unclear to me the process and the reasons for excluding the records based on relevance and design (n = 5 and n = 15 in the flow diagram). The authors should provide more detail on this. A supplementary table of the excluded studies would be helpful. It is suggested that these studies are in Supplementary Table S4, but I could not find this table.

4) The figures of the logic models contain useful information. It would be helpful to list the reference numbers for each model in the figures. Similarly, the reference numbers for each study in Table 3 should be included.

5) Several of the reference numbers are in the wrong place in the sentences and there are some references using author names (eg Armour et al at the bottom of page 4). Also, there are errors in some of the references in the reference list, such as bjog instead of BJOG.

---

## VERSION 1 - AUTHOR RESPONSE

| **Reviewer 1** | |
|---|---|
| This manuscript presents a well-conducted realist synthesis exploring how symptom-reporting tools (SRTs) are used in the identification and management of endometriosis in primary care. The methodology is appropriate, transparently reported, and aligned with RAMESES and PRISMA guidance. The inclusion of stakeholder and PPIE input is a clear strength. | We thank Reviewer 1 for the careful reading of our manuscript and for recognising the methodological rigour and the value of integrating stakeholder and PPIE perspectives throughout the synthesis. |
| However, from a clinical perspective, the manuscript remains largely explanatory and does not sufficiently clarify the implications for routine practice. While both potential benefits and harms of SRT use are described, the synthesis does not provide a clear appraisal of | We thank the reviewer for this comment and recognise the importance of clearly articulating the implications for routine practice. It is estimated that 50 million women worldwide use period tracking apps (Rosato et al 2020). Understanding how data captured through |

| | |
|---|---|
| when SRTs are likely to be helpful versus potentially detrimental. | these apps, along with any linked online resources, might influence health and patient-interactions with clinicians is therefore essential. We have included an additional section in the Discussion (Table 5) covering "considerations for clinical practice" (see page 36).<br><br>We thank the reviewer for highlighting our omission in specifying when SRT is likely to be helpful or detrimental across different contexts.<br><br>We have now addressed these points in the Discussion. Specifically, we have included a description for the contexts in which SRTs are more likely to be beneficial (e.g. consistent independent use, receptive clinicians – see lines 547 and 548) and contexts in which they may be unhelpful or harmful (e.g. when SRTs fail to capture the lived emotional impact of symptoms, when patients feel dismissed by clinicians who do not engage with the data - see lines 567 and 568). In addition, Table 4 sets out the contextual barriers and facilitators systematically across seven domains, and Table 5 translates these into practical considerations for implementation. |
| In particular, the review does not adequately consider baseline psychological vulnerability, such as anxiety or symptom hypervigilance, which are common in women with endometriosis and may substantially modify the impact of symptom tracking. Although increased distress and stress are acknowledged as possible outcomes, these factors are not explicitly integrated into the programme theories or context–mechanism–outcome configurations. | We thank the reviewer for this comment and recognise the importance of considering any baseline psychological vulnerability (e.g. anxiety, symptom hypervigilance) as a modifier of SRT impact. However, most of our included studies did not evaluate these aspects directly. The only direct evidence on this came from a small number of clinician perspectives (i.e., sexual counsellor in Oikkonen and Temmes (2025)) whose concern about heightened symptom focus through continual tracking we have incorporated as a rival theory and contextual modifier, alongside the divergent views of endometriosis nurses who generally viewed tracking positively. We have been cautious not to extrapolate beyond what the included evidence supports, in keeping with realist principles of evidence-based synthesis. But we do recognise the reviewer raised a good point and so we included the empirical question of whether and how baseline psychological factors modify SRT impact in endometriosis to our future research agenda (See page 35, Table 5 - *More research is needed on how baseline psychological factors (e.g. anxiety, symptom* |

| | |
|---|---|
| | *hypervigilance) may modify the use and impact of SRTs in) endometriosis.*<br><br>Thank you for your relevant point about considering increased distress and stress as part of our programme theories. We recognise that this is an important point. This aspect was mainly covered as part of "SRT use impact on self-management and quality of life (Section 1.3)". Evidence on increased distress and stress as part of tracking was thin and mainly supported by four studies claiming that some women experienced stress when their periods deviated from app predictions or when they forgot to enter data, which they felt undermined the accuracy of their tracking (refs 26, 31), and that for others, tracking acted as a continual reminder of ill health, contributing to reduced quality of life and increased feelings of hopelessness (refs 27, 28). In addition, our synthesis included extensive PPI and clinician input, and neither group have raised concerns about increased stress related to tracking. We also present contrasting evidence (ref 53) showing that 64% of Clue users felt they would experience more health-related stress without tracking, suggesting that for some users SRTs may reduce rather than exacerbate stress. We agree with the reviewer that this is a clear gap warranting further investigation. Therefore, we have included in Table 5 that more research is needed on whether continuous use of SRTs can increase stress and anxiety (page 35). |
| As a result, the conclusions remain theoretical and difficult to translate into clinical decision-making. The manuscript would be strengthened by: more explicit acknowledgement of the limits of clinical applicability. | We thank the reviewer for this comment, but suggest it reflects a different methodological framing than the one we adopted. Realist synthesis is designed to generate theoretical understanding of how, for whom, and in what circumstances an intervention is likely to work and it is not intended to produce prescriptive clinical decision rules or guidelines. The "theoretical" character of our conclusions is therefore a feature of the method rather than a limitation, and our findings should be read as a contribution to understanding how patients currently use SRTs, how clinicians are already engaging with them in practice, and what contextual factors shape these interactions. |

| | |
|---|---|
| | We believe this contribution is clinically useful in a different sense from clinical guidelines. The synthesis provides insight into: how women select and use SRTs across the diagnostic journey; the language and framing patients bring into consultations; how clinicians can engage productively (or unproductively) with tracker data; and the contextual conditions under which SRTs are more or less likely to support communication, referral, and shared decision-making. These insights inform tool design, primary care training, and patient-facing resources.<br><br>We do agree with the reviewer that it is important to highlight how our findings can help inform clinical decision making. Therefore, we included further considerations about implications for practice in Table 5 (page 35).<br><br>In addition, we have also clarified in the Strengths and limitations section that the predominantly cross-sectional evidence base represents perceived rather than demonstrated causal chains, and that longitudinal and prospective research will be needed before specific clinical recommendations can be made (see changes in page 32, lines 589 and 590). |
| Clearer differentiation between perceived benefits and demonstrated effects. | We thank the reviewer for this important point. Our synthesis draws on a mix of evidence types, including: quantitative behavioural data (e.g. engagement rates from the Phendo app, Elhadad et al. 2022; referral rates following PIPPA tool use, Armour et al. 2022); self-reported quantitative data (e.g. perceived knowledge improvement, Zhaunova et al. 2023; user-reported tracking outcomes, Shea et al. 2025); qualitative interview and focus group data; and validation studies (e.g. PIPPA, Parker et al. 2022). Where direct quantitative or behavioural outcomes were available, these are named explicitly in the Results with their associated figures. The language used throughout the Results ("appears to," "was reported to") reflects the predominantly cross-sectional study designs, which limit causal inference at the level of the programme theory chains.<br><br>The Strengths and Limitations section makes this explicit at the level of the synthesis as a whole, noting that "most included studies were cross-sectional, reflecting perceived rather than demonstrated causal chains" (page 32, lines 587 and 588). |

| | |
|---|---|
| Integration of mental health factors (e.g. anxiety, distress) as key contextual modifiers. | We agree that this is an important point – thank you. This was addressed above (refer to the point about distress and anxiety). See the relevant changes to the manuscript on page 35, Table 5. |
| A more explicit clinical 'bottom line' to guide primary care interpretation. | Our synthesis demonstrated substantial heterogeneity across SRTs in purpose, design, data outputs, and intended user, and a corresponding lack of standardisation in how the data are presented to clinicians. For these reasons, we do not believe a single prescriptive clinical 'bottom line' is currently supportable, and producing one was not part of our aims or objectives. |
| Overall, this is a valuable and methodologically sound contribution, but greater clarity regarding its clinical relevance and boundaries would improve its usefulness for practitioners. | We thank the reviewer once more for these constructive comments. The revisions described above together strengthen the manuscript's clinical relevance while remaining faithful to the realist methodology. |
| **Reviewer 2** | |
| This is an ambitious and comprehensive paper. It fulfils a useful role to assist the understanding of how such tools might be better developed and used in primary care. The authors have also developed a new endometriosis symptom-reporting tool, which is included in the synthesis. The results of this synthesis will inform further evaluation of this new tool. The authors provide a well-thought-out description of what a realist synthesis is and of the review's objectives. The extensive use of PPI in developing these objectives is evident in the themes' relevance to patients and clinical practice. | We thank Reviewer 2 for the generous summary of our work and for recognising the contribution of PPIE in shaping the synthesis. We address the specific points raised below. |
| The research questions did not sufficiently differentiate between a symptom tracker used to identify whether a woman might have endometriosis and one used to monitor the condition and/or when there is a need for further treatment or referral. This distinction could be quite important in the tool's application. For example, mental health effects are clearly extremely important for understanding the impact of the condition on women's quality of life. However, mental health effects may or may not be helpful in identifying who is at the highest risk of having the condition. This is particularly important when it comes to the authors' suggestion that the tool be personalised. This | We thank the reviewer for raising this important distinction. We agree with the reviewer that it is important to consider how the tracker was being used (identify endometriosis or monitor the condition) when making considerations for practice. We have amended Table 5 (page 35) to include this consideration under the “enabling personalisation of tools” section. We have also<br>Our research questions were framed broadly around the use of SRTs in primary care because, in practice, the same tool is often used across the diagnostic journey (by women who are unaware their symptoms might indicate endometriosis, those with suspected endometriosis, and those with a confirmed diagnosis). The rationale and selection strategy for the different tool types are |

| | |
|---|---|
| could be important for monitoring the condition, but not for identifying it. | described in the Methods section, with the full list of tools considered provided in Supplementary Table S4. We have also developed and tested distinct IPTs for each of the three tool types (Supplementary Figure S1), reflecting their different intended functions across identification, monitoring, and management. However, we acknowledge that current evidence did not always clearly differentiate or specify the different types of use (identification vs symptom monitoring); therefore, we have included another consideration in Table 5 under "Further research and resources" calling for further research investigating this distinction and how this can impact tool use and its effects. |
| Symptom-reporting tools are quite burdensome for patients to complete and for clinicians to interpret during a consultation. It remains unclear to me whether a system in which a woman tracks daily symptoms is preferable to a monthly symptom checklist. The burden these tools place on both patients and clinicians should be considered among the potential harms. The burden of symptom tracking and the potential for it to be harmful for some patients are illustrated in some of the quotes in the synthesis, but this needs to be highlighted more in the abstract and conclusion. | We thank the reviewer for this comment. We agree that burden is an important consideration. We have included women's accounts of stress when missing data entries, tracking acting as a continual reminder of ill health, feelings of hopelessness, and the difficulty of deciding which symptoms to prioritise within limited appointment time (see Results sections 1.3 and 2.2, and Table 4). These patient-reported burdens were given substantial weight in our synthesis and CMO configurations.<br>We have, however, been cautious not to over-amplify clinician-perceived burden in the Abstract and Conclusion, as the evidence base for this was limited and divergent e.g., endometriosis nurses generally viewed SRT data positively, while concerns about interpretation burden came from a small number of clinicians in other specialties. We felt it was important to reflect this asymmetry faithfully rather than reframe a minority clinician's concern as a main finding.<br>Regarding the comparison between daily symptom tracking and a periodic (e.g. monthly) checklist, the included evidence did not directly compare these approaches, so we are unable to draw firm conclusions on this point. However, we have acknowledged the lack of evidence comparing monthly versus daily symptom tracking in our Limitations sections; see page 34 lines 587 to 589. |
| It was unclear to me the process and the reasons for excluding the records based on relevance and design (n = 5 and n = 15 in the flow diagram). The authors should provide more detail on this. A supplementary table of the excluded studies would be helpful. It is | We apologise for the lack of clarity. Supplementary Table S4 lists the SRTs considered in the wider literature for inclusion, not the studies excluded at full-text screening.<br>To address the reviewer's request, we have now added a new supplementary table listing all |

| | |
|---|---|
| suggested that these studies are in Supplementary Table S4, but I could not find this table. | studies excluded at the full-text stage, together with the reason for exclusion (relevance, design, or other) (see Supplementary S7). |
| The figures of the logic models contain useful information. It would be helpful to list the reference numbers for each model in the figures. Similarly, the reference numbers for each study in Table 3 should be included. | Thank you for this comment. We have added reference numbers to each study row in Table 3 (pages 14 to 20) to allow readers to locate the original sources directly.<br>Regarding the logic models, we interpret the reviewer's comment as a request to indicate, within each figure, which references underpin each component or pathway. This information is provided in detail in Supplementary Table S11, which maps each CMO chain to its supporting studies. We considered adding reference numbers directly into the logic model figures but concluded that doing so would substantially reduce their readability, given the number of components and pathways involved. |
| Several of the reference numbers are in the wrong place in the sentences and there are some references using author names (e.g. Armour et al at the bottom of page 4). Also, there are errors in some of the references in the reference list, such as bjog instead of BJOG. | We thank the reviewer for these careful observations. We have undertaken a full check of the reference list and in-text citations and corrected any mistakes. |
