# Supplementary material for: Use of symptom-reporting tools to support endometriosis identification and management in primary care: a realist synthesis
Source: BMJ Open. 2026 Sep 28;16(9):e114764. doi: 10.1136/bmjopen-2025-114764 (PMC13629848; doi:10.1136/bmjopen-2025-114764)
Supplement: Supplementary file 2 [file bmjopen-16-9-s001.docx]

**Supplementary Material**

**Supplementary Table S1. SPICE Framework (Booth 2004)**

| **SPICE (criteria for search) & Supplementary information** | |
| --- | --- |
| **S**ettings | Primary care, community and domestic settings, education, online settings (websites, apps, social media) in high income countries |
| **P**erspectives | Women, girls, and those assigned female at birth (start of menarche to age of menopause) with suspected or diagnosed endometriosis, or symptoms that could be endometriosis, general practitioners, endometriosis nurses and specialists, developers of symptom reporting tools, theoretical perspectives |
| **I**ntervention/Phenomenon of Interest | Endometriosis symptom reporting tools, period symptom reporting tools with key endometriosis component or symptoms that could be endometriosis, the envelope of health information surrounding the tool (i.e., website or in-app articles), shared decision making, timely diagnosis, desired health outcomes |
| **C**omparisons | Different age groups, equity and different contexts (i.e., digital, cultural, income), women, girls and those assigned female at birth that use symptom reporting tools, compared to those who do not use symptom reporting tools, shared decision making with or without use of symptom reporting tools, knowledge of general practitioner (special interest in women’s health vs other interests), endometriosis specific symptom reporting tools compared to period reporting tools with key component of symptoms that could be endometriosis, co-production of symptom reporting tools (compared to lack of), evidence based symptom reporting tools (compared to tools that are not evidence based), affiliation with reputable organisations such as the NHS or government (compared to lack of), dissemination of tools (i.e., business or government funded) |
| **E**valuation | Realist programme theories offering explanation for how these symptom reporting tools are intended to work, realist programme theories offering explanation for why there is varied use of symptom reporting tools in primary care (to support the timely diagnosis of endometriosis and shared decision-making), preliminary implementation and practice considerations about using symptom reporting tools will be drafted, a realist evaluation framework for subsequent realist evaluation. |
| **Date limits on database searches** | Date limits of 2013-2023 were applied to ensure relevancy of information and feasibility of the search within the timeframe and resources available.  In 2013, period tracking apps were released alongside the narrative that tracking your cycle and symptoms can help women gain a sense of awareness and control over their bodies (Worsfold et al. 2021). In addition, 2013 marked a large increase in people using the internet for health information (UK Office for National Statistics 2020).  With awareness of endometriosis increasing within the medical and social spheres, ESHRE (European Society of Human Reproduction and Embryology) developed its first guideline on “Management of endometriosis” in 2013-2014, according to the thorough ESHRE methodology (Dunselman et al. 2014). |

**Supplementary Table S2. List of items required when reporting a realist synthesis (RAMESES checklist) (Wong et al. 2013)**

| **Reporting item** | | **Description of item** | **Reported on page(s)** |
| --- | --- | --- | --- |
| **Title** | | | |
| 1 |  | In the title, identify the document as a realist synthesis or review | 1 |
| **Abstract** | | | |
| 2 |  | While acknowledging publication requirements and house style, abstracts should ideally contain brief details of: the study’s background, review question or objectives; search strategy; methods of selection, appraisal, analysis and synthesis of sources; main results; and implications for practice | 2 and 3 |
| **Introduction** | | | |
| 3 | Rationale for review | Explain why the review is needed and what it is likely to contribute to existing understanding of the topic area | 4 & 5 |
| 4 | Objectives and focus of review | State the objective(s) of the review and/or the review question(s). Define and provide a rationale for the focus of the review | 5 |
| **Methods** | | | |
| 5 | Changes in the review process | Any changes made to the review process that was initially planned should be briefly described and justified | No changes were made |
| 6 | Rationale for using realist synthesis | Explain why realist synthesis was considered the most appropriate method to use | 6 |
| 7 | Scoping the literature | Describe and justify the initial process of exploratory scoping of the literature | 7 |
| 8 | Searching processes | While considering specific requirements of the journal or other publication outlet, state and provide a rationale for how the iterative searching was done. Provide details on all of the sources accessed for information in the review. Where searching in electronic databases has taken place, the details should include, for example, name of database, search terms, dates of coverage and date last searched. If individuals familiar with the relevant literature and/or topic area were contacted, indicate how they were identified and selected | 8 |
| 9 | Selection and appraisal of documents | Explain how judgements were made about including and excluding data from documents, and justify these | 9 |
| 10 | Data extraction | Describe and explain which data or information were extracted from the included documents and justify this selection | 10 |
| 11 | Analysis and synthesis processes | Describe the analysis and synthesis processes in detail. This section should include information on the constructs analysed and describe the analytic process | 9-11 |
| **Results** | | | |
| 12 | Document flow diagram | Provide details on the number of documents assessed for eligibility and included in the review, with reasons for exclusion at each stage, as well as an indication of their source of origin (e.g. from searching databases, reference lists and so on). You may consider using the example templates (which are likely to need modification to suit the data) that are provided | 10 |
| 13 | Document characteristics | Provide information on the characteristics of the documents included in the review | 11 |
| 14 | Main findings | Present the key findings with a specific focus on theory building and testing | 10-18 |
| **Discussion** | | | |
| 15 | Summary of findings | Summarise the main findings, taking into account the reviews objective(s), research question(s), focus and intended audience(s) | 19 |
| 16 | Strengths, limitations and future research directions | Discuss both the strengths of the review and its limitations. These should include (but need not be restricted to) (a) consideration of all the steps in the review process and (b) comment on the overall strength of evidence supporting the explanatory insights which emerged The limitations identified may point to areas where further work is needed | 19-21 |
| 17 | Comparison with existing literature | Where applicable, compare and contrast the reviews findings with the existing literature (e.g. other reviews) on the same topic | 19-20 |
| 18 | Conclusion and recommendations | List the main implications of the findings and place these in the context of other relevant literature. If appropriate, offer recommendations for policy and practice | 21-22 |
| 19 | Funding | Provide details of funding source (if any) for the review, the role played by the funder (if any) and any conflicts of interests of the reviewers | 23 |

**Supplementary Material S1 – Realist Synthesis**

The realist approach to synthesis is an ideal method for exploring the use of SRTs in endometriosis care, particularly in primary care settings. Unlike conventional approaches that focus solely on whether an intervention works (i.e., effectiveness reviews), realist synthesis delves into nuanced questions such as who benefits, under what circumstances, and how (Pawson et al. 2005). Realist methods (evaluation, synthesis) have been gaining traction in health research including more recently in reproductive medicine (Mehay et al. 2024; Slaghmuylder et al. 2024). The realist perspective provides a clear explanation of how and why context influences outcomes. These explanations are formulated as initial programme theories that are then tested using a range of data sources in a focused synthesis (e.g., primary studies, patient and public consultation, see Methods). By focusing on causal explanations and processes, realist synthesis offers a deeper understanding of the mechanisms underpinning intervention effects, generating insights that are evidence based and actionable. As such, findings from realist synthesis are crucial for developers and policymakers, who can integrate these insights for the optimal adaptation and implementation of interventions that meet the needs of diverse populations and settings (Pawson et al. 2005). Thus, realist synthesis recognises that observed outcomes are shaped by underlying causal processes triggered by contextual factors, conceptualised as context + mechanism = outcome or CMO chains (Rycroft-Malone et al. 2012) in the initial programme theories.

Context refers to the specific characteristics or circumstances surrounding an intervention. Context encompasses roles, interactions, and relationships, all of which can significantly influence the implementation process. For example, context could include a patient's age, whether they have a confirmed endometriosis diagnosis or are still seeking one, and the setting in which they use an SRT, independently at home or during a healthcare appointment. Context may facilitate, modify, constrain, or entirely shape how an intervention is carried out, making its consideration essential for successful application in practice (Pfadenhauer et al. 2015). A mechanism refers to the underlying processes, responses, or reasoning triggered by the intervention within a given context, leading to specific changes or effects. For example, one potential mechanism is that regularly tracking symptoms [that might indicate endometriosis] could enhance awareness of health, new symptoms or, changes in symptoms and triggers. The outcomes are the effects or changes resulting from the interaction between context and mechanisms, such as a timelier diagnosis of endometriosis or improved symptom management.

**Supplementary Material S2 -** **Patient and Public Involvement and Engagement**

The study was developed with Patient and Public Involvement and Engagement (PPIE) from an initial funding proposal (1 person with lived experience) through to the synthesis, which included 11 people with lived experience: 10 women diagnosed with endometriosis, 1 who suspected she might have the condition. One General Practitioner (GPs) and two endometriosis nurse specialists were also involved. We recruited PPI members (those with suspected and diagnosed endometriosis) from the charity Fair Treatment for the Women of Wales (FTWW). We held one virtual meeting with PPI and maintained ongoing communication via email and social media throughout the study. Additionally, we engaged (via an online event) with stakeholders from a large public sector organisation who attended an internal endometriosis support group.

PPI members and stakeholders contributed to tasks such as analysis and synthesis to address gaps and aid interpretation of findings, ensuring relevance and accessibility of outcomes based on their specific needs. They also helped identify barriers and facilitators for using SRTs in their care and provided insights on modifying or adapting identified SRTs to suit their needs. PPI played a key role in developing considerations for SRTs use in clinical practice. All activities followed the UK standards for Public Involvement (UK Standards for Public Involvement, 2019). We used the Guidance for Reporting Involvement of Patients and the Public 2 (GRIPP2) checklist (Staniszewska et al. 2017) to report our PPI activities and the Public Involvement in Research (PIRIT) tool to track impact (Newman, 2023). Contributors were provided with expenses and financial reimbursement for time spent on study activities (UK Standards for Public Involvement, 2019).

**Supplementary Table S3 - Initial list of symptom reporting tools identified in wider literature (pre-search)**

| **Symptom Reporting Tool type** | **Symptom reporting tools identified in wider literature (pre-search)** |
| --- | --- |
| *Endometriosis Specific* | - Endometrix - Endo Empowered (not on app store anymore) - Phendo - Flutter (not on app store anymore, website deleted) - Endo Diary, Bayer (Google play) - ENdi: Track your endometriosis (created by patient) - Frendo (the only endometriosis app that features a screening tool using a Patient Reported Outcome method) - Nezhat – Endometriosis Advisor - QENDO (Australia & New Zealand only IOS and google play) <https://www.jeanhailes.org.au/news/a-21st-century-response-to-endometriosis> - Living with endometriosis Kanoa Matton (IOS) - Endometriosis UK Pain and Symptom Diary - BUPA Endometriosis Symptom Diary - Centre for Young Women’s Health (Pain and Symptom tracker) <https://youngwomenshealth.org/guides/endometriosis-pain-and-symptom-tracking/> - The endometriosis foundation – symptom diary <https://www.theendometriosisfoundation.org/your-symptom-diary> - Health marketing company - <https://onyxhealth.com/healthcare-marketing-communications/endometriosis-campaign/> - <https://www.endozone.org.au/> - listed on website and can complete online no need to print (can download report) - The Raising Awareness Tool for Endometriosis (RATE) - <https://ranzcog.edu.au/resources/raising-awareness-tool-for-endometriosis-rate/> - <https://virtual.miceneurol.com/endoSurvey/KKH> - <https://endometriosis.cymru/srt/> Endometriosis Cymru Symptom Tracking and Reporting Tool (ESTR) |
| *Menstrual Pain Specific* | - Menstrual Health Project (based in Wales) –a menstrual health toolkit (can log period pain and other symptoms) designed for under 18s. They also feature an endometriosis toolkit. - <https://www.jeanhailes.org.au/resources/period-pain-symptom-diary> includes endometriosis on website and resources - PIPPA (Period ImPact and Pain Assessment) - Healthinfo.org.nz (no key endometriosis component but features on endometriosis page) - <https://hellolucy.app/en/> - Lucy |
| *Generic period trackers & chronic illnesses* | - Flo (230 million) - Clue (10 million people in over 190 countries**)** - Period Tracker Period Calendar. Used by over 300 million women, recommended by endometriosis patient via blog - Eve by Glow – Period Tracker, <https://themighty.com/topic/endometriosis/best-period-tracking-apps-endometriosis/> 33753 endometriosis members & Glow (older version still in use) - Myflo - Life – period tracker (apple app store) - [**https://thehappypelvis.ca/free-pelvic-pain-symptom-tracker/**](https://thehappypelvis.ca/free-pelvic-pain-symptom-tracker/) Used by endometriosis patients / can be used for pelvic pain other than endometriosis - <https://careclinic.io/endometriosis-symptom-tracker/> - according to website largest symptom tracking app for many conditions not just endometriosis. |

**Supplementary Figures S1.a to S1.g**

**Supplementary Figure S1.a** Logic Model illustrating the programme theory for Flo

**Supplementary Figure S1.b** Logic Model illustrating the programme theory for Period Impact and Pan Assessment (PIPPA)

**Supplementary Figure S1.c** Logic Model illustrating the programme theory for the RATE Tool

**Supplementary Figure S1.d** Logic Model illustrating the programme theory for Phendo

**Supplementary Figure S1.e** Logic Model illustrating the programme theory for Frendo

**Supplementary Figure S1.f** Logic Model illustrating the programme theory for Menstrual Health Project

**Supplementary Figure S1.g** Logic Model illustrating the programme theory for Endometriosis Cymru Symptom Tracking and Reporting Tool (ESTR)

**Supplementary Figure S2.** Logic Model illustrating the initial programme theories. Key – SRT: Symptom Reporting Tool

**Supplementary Table S4.** Programme Theories of Symptom Reporting Tools as part of endometriosis identification and management in primary care

| **Initial Programme Theory** | **Context-mechanism-outcomes** (CMO) |
| --- | --- |
| **Unaware symptoms could be Endometriosis** | **Women Perspective**    **IF** women who are unaware their symptoms are not normal, and could be related to endometriosis, use symptom reporting tools to track their cycles and associated symptoms generally, and learn more about their body / female health **(context), THEN** this helps them to communicate better about their health experiences when needed **(mechanism); LEADING TO** timelier diagnosis of conditions like endometriosis **(outcomes)**. |
|  | **Health Care Professional (Primary Care) Perspective**    **IF** healthcare professionals in primary care consult with *women who are not aware their symptoms are not normal and could be related to endometriosis,* who are using period symptom reporting tools and sharing their Symptom Reporting Tool data to improve communication with healthcare professionals during consultations; **THEN**  healthcare professionals are better informed and able to involve the patient to better tailor treatment pathways, aiding in more timely management of symptoms and better-informed referrals and further examinations; **LEADING TO** more timely diagnosis of underlying pathology such as endometriosis, improved healthcare professional-Patient relationships going forward, better patient outcomes, improving continuity of care, and reduced utilisation of primary care. |
| **Suspected** | **Women Perspective**    **Theory 1**    **IF** women who suspect something is wrong use symptom reporting tools to track their period pain and associated menstrual symptoms, this in turn improves their knowledge of what is and is not normal menstruation and they learn about possible causes for their symptoms such as gynaecological illnesses **(context).** This increased knowledge helps them to know what symptoms to look out for in considering different illnesses, and when/why to seek help **(mechanism).** This leads to more timely diagnosis of conditions like endometriosis **(outcomes).** This also leads to improved communication with healthcare providers because the person is becoming more familiar with their body and has collected data to support their experiences which could also support shared decision making **(outcomes)**.    **Theory 2**    Women using symptom reporting tools for suspected endometriosis, improve their ability to recognise and understand symptoms that could be endometriosis **(context)**, which enables them to feel validated in their health experiences and more empowered to communicate and actively engage within their care **(mechanism)** which in turn:     1. Increases patient activation from the offset of the specific care journey, leading to improved communication and thus shared decision making with their healthcare providers **(outcomes)**      2. Reduces time between symptom reporting and receiving care, streamlines symptom reporting thus helping to reduce repeat consultation, testing and treatment, leading to more timely diagnosis of endometriosis **(outcomes)** |
|  | **Healthcare Professional (Primary Care) Perspective**    **Theory 1**  **IF** healthcare professionals in primary care consult with patients who *suspect something is wrong* and they are using symptom reporting tools [for period/pelvic pain] which improves communication and explains their symptoms [providing metrics and evidence via the app]; **THEN** healthcare professionals can create shared goals with the patient regarding the next steps in their care which could involve more streamlined investigations, tests, referrals, further education, and symptom management; **LEADING TO** more timely diagnosis and management of endometriosis, thus better outcomes for the patient and less burden on health services.    **Theory 2**  **IF** healthcare professionals in primary care consult with patients using symptom reporting tools for *suspected endometriosis* to explain symptoms patients think could be related to endometriosis [providing metrics and evidence via the app]; **THEN** healthcare professionals can create shared goals with the patient regarding the next steps in their care which could involve more streamlined investigations, tests, referrals, further education, and symptom management; **LEADING TO** more timely diagnosis and management of endometriosis, thus better outcomes for the patient and less burden on health services.    **Potential rival theory –** Symptom Reporting Tools hinders communication affecting further outcomes |
| **Confirmed Endometriosis** | **Women Perspective**    Women using symptom reporting tools to track confirmed endometriosis, improve their ability to recognise and understand the condition and how it affects them **(context)**, which enables them to know how to better self-manage and when / how to communicate with their health care professionals about new and/or unmanaged symptoms **(mechanism)**, which leads to improved shared decision making and better care outcomes on an ongoing basis **(outcomes).** |
|  | **Healthcare Professional (Primary Care) Perspective**    **IF** healthcare professionals consult with patients who use symptom reporting tools to monitor/track *confirmed endometriosis* and share their data during consultations to improve communication and facilitate the identification of new or changes in symptoms; **THEN** shared decision making is improved with the creation of share goals thus enabling more timely and tailored management of condition, prioritisation of urgent needs and more timely and informed referrals; **LEADING TO** better health outcomes for patients and a more joined up care approach that relieves primary care services**.** |

**Supplementary Table S5. Final Search Strategies Used**

Our primary search was conducted using Google Scholar and supplemented by searches in Press Reader, PubMed, Web of Science, LibrarySearch, APA PsycINFO via Ovid, and Scopus. Grey literature was sought via search engines, databases such as Overton, and from key stakeholders. Searches for grey literature included endometriosis-related documents (policy papers, guidelines), reports, blogs, newspapers, and magazines. Usually, documents for realist searches are not designed to be exhaustive and usually include a wide range of sources with no restriction to document type (Booth, Briscoe, and Wright 2020).

In accordance with RAMESES guidance (Wong et al. 2013) and established practice for realist syntheses (Booth, Briscoe and Wright 2020), our search was purposive, iterative, and theory-driven rather than a single locked strategy applied uniformly across databases. The core search concepts shown below were developed during initial scoping, adapted to the syntax of each database (Boolean operators, proximity operators, and subject headings where available), and revisited iteratively throughout the synthesis as programme theories were tested and refined. Searches were conducted between January and April 2024 and updated in October 2025.

**Filters and limits.** English-language sources only; no document-type restrictions were applied, consistent with realist searching, which draws on peer-reviewed studies, grey literature, policy documents, blogs, news media, and social media. Searches were limited to sources published from 2013 onwards. As the convergence of widely available tracking tools, online health-information seeking, and formal clinical guidance on endometriosis dates from this period, a 2013 start date captures the relevant evidence base.

**Core search concepts.** Terms within each block were combined using AND/OR to form our search strings, which were adapted to the syntax of each platform.

| *1.* | **Endometriosis**  Endometriosis OR Dysmenorrhea OR (painful adj3 menstruation) OR (painful adj period*) OR (menstrual adj disorder*) OR (period adj3 pain) OR (menstrual adj health) OR Menstrua* |
| --- | --- |
| *2.* | **Symptom-reporting tools**  Symptom eval* OR symptom report* OR smartphone OR mobile OR mhealth OR screen* OR tool OR track* OR diar* OR journal OR questionnaire OR survey OR app* OR self-manag* OR self-assess* OR self-report* OR (patient adj report*) OR Website OR Webpage OR Online OR Internet OR (social adj media) OR (information adj seeking) OR (web-based adj2 resource) |
| *3.* | **Timely Diagnosis**  Timely diagnosis OR barriers OR facilitators OR (late adj3 diagnosis) OR (delay* adj3 diagnos*) OR (early adj3 diagnosis) |
| *4.* | **Shared-decision making**  Shared decision making OR person-cent* care OR (decision adj3 support) OR patient perspective* OR GP perspective* OR Healthcare provider* perspective* OR (health adj literacy) OR patient activation OR decision aid* OR aid* decision OR decision OR patient-cent* OR communication |

This strategy was supplemented through additional searches utilising a combination of the following keywords, linked by ‘OR’/’AND’ (Bramer et al. 2018): endometriosis; timely diagnosis; shared decision making; SRTs; websites; symptom tracking; period tracker; internet; mobile app; painful periods; painful menstruation; menstrual disorders; menstrual trackers; webpages; online; social media; diary; diaries; delayed diagnosis; health information; screening tool; dysmenorrhea.

Searches were further supplemented by berry-picking and CLUSTER searching techniques (Tsang and Maden 2021) used in realist methods that involved:

- Checking reference lists from key studies and systematic reviews (snowballing)
- Citation searches (forwards and backwards)
- Consulting with stakeholders and PPI groups (regarding endometriosis policy documents, grey literature, and other sources such as social media or magazine articles etc.)
- Identifying kinship or sibling studies

**Supplementary Table S6.** Characteristics of included studies and sample (n = 19)

| **Authors (year)**  *Study Settings* n (%) | **Study Design** | **Symptom Reporting Tool (SRT)** | **Population,**  *Total n (%)* | **Age** (range) | **Gender or Sexual Orientation** n (%) | **Ethnicity/Race** n (%) | **Education** n (%) |
| --- | --- | --- | --- | --- | --- | --- | --- |
| Avery et al (2022) Armour,  *Australia* | Commentary | RATE, PIPPA | N/A | N/A | N/A | N/A | N/A |
| Armour, Parry et al (2022)  *Australia* | Pre-post single group evaluation design | PIPPA | Confirmed endometriosis: 18 (24%)  Suspect something is wrong: 55 (73%)  Unaware: 2 (3%)  *Total (n) = 75* | 14–25 | N/R | N/R | School or university: (60%) |
| Broad, et al (2022) (Broad,  *United Kingdom: (91.5%)*  *Other: (8.5%)* | Cross-sectional Mixed-methods Observational Survey | N/R | Confirmed and Undiagnosed (not specified): 330 (100%)  *Total (n) = 330* | 14-54 | Heterosexual: 287 (87%)  Homosexual: 3 (0.9%)  Bisexual: 30 (9.1%)  Pansexual: 5 (1.5%)  Asexual: 2 (0.6%)  Prefer not to say: 3 (0.9%) | White-British: 241 (73.3%)  White-Irish: 11 (3.6%)  Other White background: 34 (10.6%)  Black/Black-British – African: 5 (1.8%)  Black/Black-British – Caribbean: 3 (0.9%)  Asian/Asian British – Indian: 9 (3%)  Other Asian background: 6 (2.7%)  Latino: 3 (1.2%)  Arab: 1 (0.6%)  Mixed ethnic background: 13 (4.2%)  Other: 1 (0.6%)  Prefer not to say: 1 (0.3%) | Secondary school: 5 (1.5%)  College level: 63 (18.8%)  University undergraduate: 140 (42.2%)  University postgraduate: 115 (34.9%)  Other: 7 (2.4%) |
| Elhadad, et al (2022) [preprint]  *United States* | Longitudinal Observational Cohort study | Phendo | Confirmed diagnosis: 3136 (62.8%)  Self-suspected: 1060 (21.2%)  Provider suspected: 797 (16%)  *Total (n) = 4993* | 13-62 | Woman: 4877 (97.7%)  Man: 7 (0.1%)  Other: 69 (1.4%)  No answer: 40 (0.9%) | White, non-Hispanic: 4060 (81.3%)  Other: 311 (6.2%)  Hispanic: 283 (5.7%)  Black, non-Hispanic: 137 (2.7%)  Asian: 130 (2.6%)  Native American: 29 (0.6%)  No answer: 43 (0.9%) | College: 2902 (58.1%)  Some College: 1428 (28.6%)  High School Graduate or undergraduate: 616 (12.3%)  No answer: 47 (0.9%) |
| Frayne, et al (2023)  *Australia* | Cross-sectional Mixed-Methods Pilot | RATE | General Practitioners: 12 (9%)  Patients: 111 (91%); *among these, 8* (7.8%) were *diagnosed with endometriosis*  *Total (n) = 123* | General Practitioners: 30-61  Patients: 18-50 | Woman: 2  Man: 10 | N/R | N/R |
| Ford, De Togni, and Miller (2021)  *United States* | Cross-sectional Qualitative | N/R | App users (undiagnosed): 6 (100%)  *Total (n) = 6* | 19-35 | Woman: 5 (83.3%)  Non-binary: 1 (16.7%) | Euro-Americans: 4 (66.6%)  Middle Eastern American: 1 (16.7%)  Asian American: 1 (16.7%) | Affiliated with higher education institutions: 6 (100%) |
| Hohmann-Marriott, Williams, and Girling (2023)  *New Zealand* | Cross-sectional qualitative exploratory | Most common SRT were ‘Flo’ (51%), Clue (30%), Others (14%) | Health care professionals: 36 (25%)  Patients: 89 (62%)  Not identified: 19 (13%)  *Total (n) = 144* | **Health care professionals:**  22-50  **Patients:**19-55 | **Health care professionals:**  Woman: 35 (96%)  **Patients:**  Woman: 87 (98%) | **Health care professionals:**  New Zealand European: 116 (81%)  Māori: 8 (6%)  Other: 20 (14%)  **Patients:**  European: 125 (87%)  Māori: 8 (6%)  Pacific peoples: 3 (2%)  Other: 8 (6%) | N/R |
| Hohmann-Marriott, et al (2021)  *New Zealand* | Cross-sectional Qualitative study | N/R | Undiagnosed (not specified): 25 (100%)  *Total (n) = 25* | 18-39 | All identified as Woman | New Zealand European: 24 (96%)  Māori: 1 (4%) | Participants had relatively high levels of education, ranging from secondary to postgraduate |
| Hong, et a (2024)  *South Korea* | Cross-sectional Mixed-Methods study | Period Tracking Apps (e.g., Pink diary, Apple Health and Samsung Health) | Undiagnosed (not specified): 692 (100%)  Total (*n*) = 692 | 20-39 | N/R | N/R | High School: 56 (8.1%)  College 138 (19.9%)  University: 450 (65%)  University postgraduate: 48 (6.9% |
| Levy and Romo-Avilés (2019)  *Austria, Spain and Italy* | Cross-sectional qualitative study | N/R | Undiagnosed: 26 (100%)  *Total (n) = 26* | 18-40 | Woman: 24.96 (96%)  Non-binary: 1 (4%) | White: 25 (96%)  Non-white: 1 (4%) | Apprenticeship: 4 (15%)  High school diploma: 4 (15%)  Bachelor's or Master's degree: 14 (54%)  Doctorate degree: 4 (15%) |
| McKillop, et al (2016)  *United States* | Cross-sectional mixed methods | N/R | Confirmed endometriosis: 27 (100%)  *Total (n) = 27* | 27-60 | All woman (gender not specified) | N/R | N/R |
| Oikkonen and Temmes (2025)  *Finland* | Qualitative Case study | Endometriosis-specific app developed by a  patient organisation | Confirmed endometriosis: 59 (77.5%)  Health care professionals: 13 (17.5%)  Patient organisation (app developers): 4 (5%)  Total (n) = 76 | N/R | Mostly cisgender women | N/R | N/R |
| Parker, et al (2022)  *Australia* | Cross-sectional Quantitative Survey | PIPPA | Undiagnosed: 981 (92%)  Uncertain about diagnosis: 54 (5%)  Symptoms that could be endometriosis: 10 (1%)  Diagnosed with laparoscopy: 10 (1%)  *Total (n) = 1066* | 15-19 | N/R | N/R | Senior high school: 1066 (100%) |
| Patel, et al (2024)  *United Kingdom: 89%*  *Other: 11%* | Observational mixed-methods study | Clue (38.8%, 50/129) and Flo (29.5%, 38/129) were the most popular choices | Undiagnosed (not specified whether participants suspected something wrong or suspected endometriosis) & Diagnosed: 326 (100%)  *Total (n) = 326* | 14-54 | Heterosexual: 276 (84.4%)  Homosexual: 4 (0.9%)  Bisexual: 30 (8.9%)  Pansexual: 6 (1.5%)  Asexual: 3 (0.6%)  Prefer not to say: 3 (0.9%) | White-British: 226 (69.3%)  White-Irish: 8 (2.5%)  Any other white background: 34 (10.4%)  Black/Black British African: 6 (1.8%)  Black/Black British- Caribbean: 2 (0.6%)  Any other Black/African/ Caribbean background: 1 (0.3%)  Asian/Asian British- Indian: 8 (2.5%)  Asian/Asian British Pakistani: 3 (0.3%)  Any other Asian background: 8 (2.5%)  Mixed ethnic background: 14 (4.3%)  Arab: 2 (0.6%)  Latino: 4 (1.2%)  Prefer not to say: 12 (3.7%) | Secondary school: 5 (1.5%)  University undergraduate: 135 (41.4%)  University postgraduate: 108 (33.1%)  Other: 8 (2.5%)  College: 61 (18.7%) |
| Peven, et al (2023)  *United Kingdom* | Cross-sectional Clinical Vignettes Study | Flo | General practitioners: 16 (100%)  *Total (n) = 16* | N/A | N/A | N/A | N/A |
| Pichon, et al (2021)  *United States* | Cross-sectional Qualitative study | Not reported | Healthcare professionals: 10 (32%)  Confirmed endometriosis patients: 21 (68%)  *Total (n) = 31* | **Patients:** 21-41 | **Healthcare professionals:**  Woman: 7 (22.5%)  Man: 3 (9.7%)  **Patients:**  Women: 21 (67.8) | **Patients:**  White: 14 (67%)  Black: 5 (24%)  Latina: 2 (10%)  Asian: 1 (5%) | N/R |
| Scheck, et al (2023)  *New Zealand* | Cross-sectional Quantitative Survey | Most commonly used were ‘Flo’  (31.1%), and ‘Clue’ (24.6%) | Confirmed Endometriosis: 125 (66.5%)  Suspected Endometriosis: 63 (33.5%)  *Total (n) = 188* | 16-47 | Woman: 178 (94.7%)  Man: 2 (1.1%)  Non-binary, gender or another gender: 8 (4.3%) | Māori: 23 (12.2%)  Pacific: 4 (2.1%)  New Zealand European: 162 (86.2%)  Other: 21 (11.2%) | N/R |
| Shea, et al (2025) [preprint]  *Across 136 countries* | Cross-sectional Quantitative Survey | Clue | Confirmed Endometriosis: 547 (5%)  Total (*n*) = 11,910 | 18–58 | N/R | White: 8,637 (75%)  Black: 728 (6%)  Asian: 1,032 (11%)  Latinx: 1,309 (9%)  Arab: 144 (1%)  Other: 564 (5%) | Primary/elementary school or less: 77 (1%)  Secondary/high school or equivalent: 3,307 (28%)  Trade School: 546 (5%)  University: 5,102 (43%)  Postgraduate: 2,878 (24%) |
| Zhaunov, et al (2023)  *United States: 758 (34.3%)*  *United Kingdom: 217 (9.8%)*  *Canada: 121 (5.5%)*  *India: 108 (4.9%)*  *Australia: 92 (4.2%)*  *South Africa: 86 (3.9%)*  *Nigeria: 82 (3.7%)*  *Ghana: 63 (2.8%)*  *Philippines: 43 (1.9%)*  *Netherlands: 32 (1.4%)* | Cross-sectional Quanitative Survey | Flo | *Total (n) = 2 212* | 18-54 | Woman: 2139 (96.7%)  Nonbinary: 22 (1%)  Genderqueer or gender fluid: 19 (0.8%)  Questioning or unsure: 10 (0.4%)  Man: 5 (0.2%)  Trans man: 3 (0.1%)  Trans woman: 1 (0.1%)  Agender: 3 (0.1%)  Prefer not to say: 11 (0.5%) | White, European American, or Caucasian: 1041 (47%)  Black or African American: 397 (17.9%)  Asian or Asian American: 223 (10.1%)  Hispanic, Latino, Spanish origin: 116 (5.2%)  Biracial or multiracial: 88 (4%)  American Indian or Alaskan: 12 (0.5%)  Native Hawaiian or Pacific Islander: 3 (0.1%)  Other: 225 (10.2%)  Prefer not to say: 107 (4.8%) | Doctorate degree: 43 (1.9%)  Master’s degree: 373 (16.9%)  Bachelor’s degree: 759 (34.3%)  Associate’s degree: 139 (6.3%)  High school graduate or diploma: 600 (27.1%)  Some high school, no diploma: 115 (5.2%)  Prefer not to say: 112 (5.1%)  Other: 71 (3.2%) |

Key - N/A: non-applicable; N/R – not-reported; PIPPA: Period ImPact and Pain Assessment tool; RATE: Raising Awareness Tool for Endometriosis.

**Supplementary Table S7. References of all studies excluded at full-text screening, with reasons for exclusion**

| **References of excluded studies** | **Reasons for exclusions** |
| --- | --- |
| Ensari, I., Horan, E., Elhadad, N., & Bakken, S. R. (2022). Evaluation of a disease-specific mHealth-based exercise self-tracking measure. medRxiv [Preprint]. | Focuses on exercise self-tracking; does not address use of the tool of interest in practice. |
| Ensari, I., Lipsky-Gorman, S., Horan, E. N., Bakken, S., & Elhadad, N. (2022). Associations between physical exercise patterns and pain symptoms in individuals with endometriosis: a cross-sectional mHealth-based investigation. BMJ Open, 12(7), e059280. | Mentions Symptom Reporting Tool (SRT) (i.e., Phendo), but examines the association between exercise and pain symptoms rather than the tool's use in practice. |
| Özcan, H., Burger, N. B., van Dulmen-den Broeder, E., van Baal, M. W., van den Boogaard, E., De Leeuw, R. A., & Huirne, J. A. F. Instruments to identify menstrual complaints and their impact on adolescents: a systematic review. J Pediatr Adolesc Gynecol. 2024 Apr;37(2):106-120. doi: 10.1016/j.jpag.2023.11.011. Epub 2023 Dec 16. PMID: 38104798. | Mentions SRT (i.e., PIPPA), but does not discuss its use. |
| Urteaga, I., McKillop, M., & Elhadad, N. (2020). Learning endometriosis phenotypes from patient-generated data. npj Digital Medicine, 3(1), 88. | Mentions SRT (i.e., Phendo), but focuses on phenotyping rather than the instrument's use in practice |
| Urteaga, I., McKillop, M., Lipsky-Gorman, S., & Elhadad, N. (2018). Phenotyping endometriosis through mixed membership models of self-tracking data. arXiv [Preprint]. arXiv:1811.03431. | Mentions SRT (i.e., Phendo), but focuses on phenotyping from the authors' own data rather than the instrument's use in practice. |

**Supplementary Table S8. Symptom Reporting/Tracking tools named in the included studies**

| **NAME** | **TYPE** | **MODE OF COMPLETION** | **MEASURE/FUNCTION** |
| --- | --- | --- | --- |
| [**FLO**](https://flo.health/) (Grieger JA 2020; Cunningham AC 2024)  https://flo.health/ | Generic Period Tracker | Mobile app. Available on app stores, worldwide. | Flo is among the world’s most popular period trackers, offering features to track periods, ovulation, fertility, and pregnancy. This Symptom Reporting Tool has an extensive health library including evidence-based information on various topics, including menstrual health disorders (i.e., endometriosis), and general menstrual health. Flo offers both a free version and a premium paid version. |
| [**Period ImPact and Pain Assessment (PIPPA)**](https://www.canberrahealthservices.act.gov.au/services-and-clinics/services/canberra-endometriosis-centre/period-impact-and-pain-assessment-pippa) (Mike Armour 2021; Parker et al. 2022)  https://www.canberrahealthservices.act.gov.au/services-and-clinics/services/canberra-endometriosis-centre/period-impact-and-pain-assessment-pippa/period-impact-and-pain-assessment-pippa-online-screening-tool | Menstrual Pain Specific | Web-based. Embedded in Canberra health service’s website. | PIPPA is an evidence-based tool that assesses period pain via a 5-item survey. This tool provides evidence-based information on menstruation and self-management strategies, along with additional resources. PIPPA also provides insights into the relevance of the users’ PIPPA score for healthcare providers. Translation options are available. |
| [**The Raising Awareness Tool for Endometriosis (RATE)**](https://ranzcog.edu.au/resources/raising-awareness-tool-for-endometriosis-rate/%22%20/l%20%22waypoint=about-rate)  (Frayne et al. 2023)  https://ranzcog.edu.au/resources/raising-awareness-tool-for-endometriosis-rate/ | Endometriosis Specific | Web-based. Embedded in the Royal Australian and New Zealand College of Obstetricians and Gynaecologist’s website. | RATE is an evidence-based tool designed to facilitate conversations with clinicians about symptoms possibly related to endometriosis. It features 16 questions, evidenced-based information on symptoms, and external resources. It also includes a section for the user to note questions for their doctor. The questionnaire responses and questions are summarised in an online report, accessible to the user via email. |
| [**PHENDO**](https://citizenendo.org/) (McKillop, Mamykina, and Elhadad 2018)  https://citizenendo.org/ | Endometriosis Specific | Mobile app. Available on app stores, worldwide. | Phendo is a research app that allows women to track, understand and manage endometriosis. This Symptom Reporting Tool is freely available and has over 15,000 users. Phendo aims to understand people’s experiences with endometriosis and the various phenotypes of the condition. |
| Clue: Period & Cycle Tracker for iOS and Android  https://helloclue.com/ | Generic Period Tracker | Mobile app. Available on app stores, worldwide. | Clue is a women-led generic period tracker, available in more than 20 languages and developed to track fertility, period, and mood. |

**Supplementary Table S9.** Engagement, Acceptability, Sensitivity, Specificity, and Effects of Symptom Reporting Tools (n = 19)

| **Authors, year** | **Study Aims** | **Symptom reporting tool used** | **Tool engagement and/or acceptability** | **Sensitivity/**  **specificity** | **Outcomes and consequences of symptom tracking** |
| --- | --- | --- | --- | --- | --- |
| Armour, Avery et al (2022) | [Commentary] | RATE, PIPPA | N/A | N/A | Use of screening tools in young women for early screening of menstrual disturbances and a stepped pathway for early management of painful periods and possible endometriosis |
| Armour, Parry et al (2022) | This study aimed to assess the feasibility and acceptability of a web-based resource for improving menstrual health literacy and encouraging health-seeking behaviour among individuals under 25, who often self-manage conditions like dysmenorrhea and heavy bleeding despite their impact on daily life. | PIPPA | Participants rated the tool as acceptable. Eighty-five percent of participants found the web resource easy to use, and 90% considered the information "very helpful." Engagement  with the resource decreased slightly over time, from 97% at baseline, 89% at  month one, 79% at month two, and 75% at month three | N/A | The tool changed the way 60% of  participants managed their menstrual symptoms, with 85% continuing to use at least one of the  self-management practices from the tool. Forty-eight percent revised their understanding of a "normal" period, and 43% consulted a doctor about menstrual symptoms, with 84% doing so because of the resource. Of those, 56% received a referral to a gynaecologist |
| Broad, et al (2022) | This study aimed to explore women's real-life experiences with period tracker apps, their attitudes toward using them, the information provided on ovulation, and how the accuracy of period predictions influenced their feelings and behaviours. | Not reported | N/A | N/A | Most respondents had a positive experience, with the top three feelings being 'Prepared' (53.3%), 'In control' (49.0%), and 'Informed' (36.7%). 'Indifferent' was selected by 16.7%, while the most common negative feeling was 'Frustrated' (4.8%) |
| Elhadad, et al (2022) | This study examined engagement patterns in Phendo, an endometriosis self-tracking app, analysing short-term (first 12 weeks) and long-term usage across different tracking domains. The study then evaluated long-term engagement from onboarding to the last recorded interaction, including disease-specific tracking behaviours. | Phendo | The study identified four participant groups in the Phendo cohort based on short-term engagement: Regulars, Usuals, Occasionals, and Seldoms. These groups, differing only in age and education, maintained distinct long-term engagement patterns. Regulars were more likely to use self-tracking for disease management | N/A | N/A |
| Frayne, et al (2023) | This study aimed to evaluate the acceptability of the Raising Awareness Tool for Endometriosis (RATE) among general practitioners (GPs) and assess the prevalence of symptoms in women presenting to general practice, along with their immediate management. | RATE | Patients considered the RATE tool as acceptable for use in general practice settings. | N/A | The tool helped to identify symptoms and initiated discussions on potential diagnoses and management of endometriosis. After symptoms were identified, GPs arranged individualised investigations and follow-up. Further GP education is needed to identify women at the highest risk of developing chronic pain syndromes |
| Ford, et al (2021) | This study investigated how managing biometric menstrual data shapes individuals' relationships with their bodies and hormonal cycles. The study explored app usage, menstrual experiences, gender identity, perceptions of hormones, data privacy concerns, and views on health and self-care, allowing participants to highlight what mattered most to them. | Not reported | N/A | N/A | Women used Symptom Reporting Tools to understand and manage experiences associated with their periods, particularly changes in mood |
| Hohmann-Marriott, et al (2023) | This exploratory qualitative study aimed to understand how healthcare providers, patients, and app users perceive menstrual apps. | Most common Symptom Reporting Tool were ‘Flo’ (51%), Clu (30%), Others (14%) | N/A | N/A | Symptom-tracking provided a reliable and credible way of recording symptoms and cycle dates, which improved communication between patients and healthcare providers. However, participants felt that these apps are limited in their ability to help and called for more assistance in managing menstrual disorders and diseases (e.g., more effective treatment and management options). |
| Hohmann-Marriott, et al (2021) | This study explored menstrual app users' experiences and perceptions, focusing on menstrual and digital health. It examined how users describe the data they enter and any concerns they have about their data and information. | Not reported | N/A | N/A | Women generally perceived their data as highly valuable and found tracking their menstrual cycles to be an empowering experience |
| Hong, et a (2024) | This study explored how menstruation-related experiences shape, and are shaped by, the use of mobile period-tracking apps among millennial and Generation Z individuals. | Not reported | N/A | 46.2% women reported SRT predicted their cycle with a 2-day error range more than 10 times a year. Although this was not the case for those with irregular cycles. | SRT use was associated with greater body awareness, leading to positive social, academic, and health-related outcomes. |
| Levy, et al (2019) | This qualitative study provided insight into users' practices and experiences with app-supported menstrual tracking. | Not reported | N/A | N/A | Symptom Reporting Tools increased women’s awareness of their menstrual cycles and health. Menstrual tracking can lead to distress in some cases, to privacy issues and increase burden on women |
| McKillop, et al (2016) | The study sought to explore women's attitudes and motivations toward self-tracking their experience of endometriosis and assess the feasibility of using self-tracking for phenotyping the disease. | Not reported | Recently diagnosed endometriosis patients were more eager to use self-tracking to understand changes in their endometriosis symptoms. Perceptions toward existing self-tracking apps were negative and women felt that these apps did not capture their experiences of endometriosis | N/A | Symptom Reporting Tools were used not only to manage endometriosis symptoms but also to manage quality of life. Participants described self-tracking as useful for documenting symptoms and symptom patterns. Self-tracking was also seen to “educate” providers about patients’ experiences |
| Oikkonen, et al (2025) | The study examined an endometriosis-specific app developed by a patient/advocacy group. It explored how well the app captured the specificities of endometriosis, assessed its technological and structural features, and analysed how different user groups engaged with it. | Endometriosis-specific app developed by a patient/advocacy group | N/A | N/A | The app was seen to support detailed, condition-specific symptom tracking, enabling users to identify changes and treatment effects that fall outside the expectations of a typical menstrual cycle. |
| Parker, et al (2022) | This study aimed to validate the PIPPA self-screening tool for menstrual disturbance in teenagers by evaluating its performance against the MDOT questionnaire. | PIPPA | N/A | Use of PIPPA scores had good predictive accuracy for predicting MDOT (Menstrual Disorder of Teenagers) diagnosis of endometriosis (area under the curve [AUC] of  0.789); school absences (AUC of 0.813); severe pain (AUC of 0.855) | N/A |
| Patel, et al (2024) | This study explored period tracking app users' experiences, including which apps they used, their frequency of use, the types of data they tracked, and their attitudes toward these apps. | Clue (38.8%, 50/129) and Flo (29.5%, 38/129) were the most popular choices | Most participants (61.9%) used a period tracking app for 2 years or more. Of these, 85.6% recorded more data during their period, with 31% doing so once a day. | N/A | Most participants (83.1%, 271 out of 326) felt that using a period-tracking app improved their understanding of their menstrual cycle. However, the majority (69.9%, 228 out of 326) only had partial trust in the information the app provided |
| Peven, et al (2023) | This study aimed to evaluate the agreement between clinicians and three Flo Health UK symptom checkers in assessing endometriosis, uterine fibroids, and Polycystic ovary syndrome using vignettes. It also aimed to provide a robust example of vignette case creation, review, and classification for pre-deployment testing and validation of digital health symptom checkers. | Flo | High levels of agreement between general practitioner and symptom checker 83.3% | Sensitivity of symptom checker for endometriosis (81.8%), Specificity of symptom checker for endometriosis (84.6%) | N/A |
| Pichon, et al (2020) | This study explored how women with endometriosis managed their condition independently and with healthcare providers. The study also examined the challenges of caring for a complex disease and the role of technology in supporting care and collaboration. | Not reported | N/A | N/A | Self-tracking helped patients to reflect on, and make sense of the endometriosis illness experience, plan for clinical encounters and care with treatments and self-management regimens. Providers described the complexity of reviewing and interpreting clinical and self-tracked patient data from across quite a few domains of health |
| Scheck, et al (2023) | This study examined whether women used mobile apps for symptom tracking, the symptoms they tracked, their perceived app effectiveness, and their willingness to share data with clinicians or researchers. | Most commonly used were ‘Flo’  (31.1%), and ‘Clue’ (24.6%) | 51.5% of Symptom Reporting Tool users reported logging symptoms at least weekly; 5.9% logged symptoms multiple times daily or daily (17.6%), multiple times weekly (20.6%), weekly (7.4%), multiple times per cycle (26.5%) and monthly (22.1%) | N/A | Use of Symptom Reporting Tools for symptom tracking (such as periods, pain, bowel symptoms, mental health symptoms) was reported by 83/188 (44.1%) |
| Shea, et al (2025) | The study examined how menstrual tracking apps support users’ menstrual health experiences, self-management, and clinical engagement, and assessed their benefits and limitations. | Clue | Tool engagement and/or acceptability | Clue helped women (independent of diagnose) (31%) discover a potential health issue | Outcomes and consequences of symptom tracking |
| Zhaunova, et al (2023) | This study aimed to explore knowledge and health improvements related to the menstrual cycle, pregnancy, and general health among Flo app users. It also examined which app components were linked to these improvements and whether they varied by education level, country of residence, subscription type (free or premium), duration of use, and frequency of use. | Flo | N/A | N/A | Women reported Flo improved their knowledge of menstrual cycles (89%) and pregnancy health (84%). These enhancements were mainly associated with the app's features for predicting periods and fertile days, tracking symptoms, and accessing relevant health content like articles and videos within the app.  Users who did not complete higher education degrees were more likely to report that Flo had helped them improve their understanding of how to manage their menstrual symptoms and helped them identify issues related to endometriosis. Frequent app users were also more likely to say that Flo had helped them identify issues related to endometriosis. |

Key – N/A – non-applicable; PIPPA: Period ImPact and Pain Assessment tool; RATE: Raising Awareness Tool for Endometriosis.

*Engagement, acceptability, sensitivity, specificity and effects of* *SRTs named in included studies*

Elhadad N. (2022) identified four distinct engagement patterns with the Phendo tool (Regulars, Usuals, Occasionals, Seldoms). Similarly, Patel et al. (2024) described that although most participants used period-tracking apps for over two years (N=222/326), 69.9% expressed only partial trust in the data. Armour, Parry, et al. (2022) reported high initial engagement with the PIPPA tool (N=75), with 85% finding it easy to use and 90% finding the information helpful, though usage declined from 97% at baseline to 75% at three months. Frayne et al. (2023) found the RATE tool acceptable in general practice, supporting symptom discussions and follow-up care.

SRTs were commonly used to monitor pain, bowel issues, and mental health concerns (Scheck et al. 2023; Pichon et al. 2021). They supported symptom management in endometriosis (McKillop M 2016) and menstrual health, with 60% of women in one study reporting changes in how they managed symptoms Armour, Parry, et al. (2022). Benefits included increased awareness and improved communication with healthcare providers (Elhadad N. 2022; Hohmann-Marriott, Williams, and Girling 2023), though limitations were also noted—such as inadequate reflection of lived experiences, limited management options, and privacy concerns (McKillop M 2016; Elhadad N. 2022; Hohmann-Marriott, Williams, and Girling 2023).

Regarding cycle-related health issues, Shea (2025) described users of Clue as ‘no known concern’, ‘suspected’ and ‘known condition’. Among those with concerns, 31% reported that RT use helped identify a potential health issue. In addition, Parker et al. (2022) reported that the PIPPA tool had good accuracy for identifying endometriosis-related outcomes (AUC=0.789-0.855), while Peven et al. (2023) found Flo’s endometriosis symptom checker had 81.8% sensitivity and 84.6% specificity. Frayne et al. (2023) similarly found the RATE tool useful in identifying symptoms and supporting diagnostic conversations.

 **Supplementary Table S10.** Factors creating barriers and facilitators for the use of Symptom Reporting Tools (SRTs) for endometriosis

| **Factors** | **Barriers** | **Facilitators** |
| --- | --- | --- |
| **Factors relating to tool design, functionality and accessibility** | Popular menstrual apps were often heteronormative and overtly feminine, thereby excluding the realities of many users (Epstein et al. 2017; Patel et al. 2024).  Popular menstrual apps often overlooked users with irregular cycles or those not menstruating but still interested in tracking symptoms. Period predictions commonly catered to the 'normal' cycle range of 24-38 days (Patel et al. 2024; Pichon et al. 2021).  Privacy. Users reported concerns about privacy on varying levels. Some reported concerns about how their data was used in digital apps, whilst others expressed relief in using an app instead of marking their cycle on a calendar, seeing it as a more private option. Privacy concerns affected what data users logged in to the apps (Patel et al. 2024; Hohmann-Marriott 2021; Epstein et al. 2017; Hohmann-Marriott, Williams, and Girling 2023; Oikkonen and Temmes 2025).  Cost. Users of generic period apps reported concerns about cost. Although they were interested in in-app purchases, these options were unaffordable and the advertisements for them distracted them from their data input (Patel et al. 2024).  Dissemination of endometriosis-specific tools. Most users were unaware of specific tools for certain menstrual disorders [(Hohmann-Marriott, Williams, and Girling 2023); PPI].  Data input. Users often forgot to track symptoms (such as the end of a cycle) as they did not consider it a priority. They were also selective about the type of information they inputted, often only including period dates as they did not want to track everything (Hohmann-Marriott, Williams, and Girling 2023; Patel et al. 2024; Epstein et al. 2017).  Reliability of information. Generic period apps were often not informed by evidence or expert advice. The majority of these apps also do not follow NICE Evidence Standard framework (Fani Gkrozou 2019; Patel et al. 2024; Wong et al. 2021; Reema A. Karasneh 2020; Moglia et al. 2016). This impacted the users’ trust of the information provided by these apps (Patel et al. 2024).  Suitability versus need. Generic period apps often lacked sufficient information on endometriosis and other menstrual disorders. They were not always suitable for users' needs at different life stages and were difficult to tailor for personal use. For example, some offered an overwhelming number of tracking options, but none for endometriosis and mental health impacts. This resulted in fragmented tracking across multiple apps or disengagement [(Hohmann-Marriott, Williams, and Girling 2023; McKillop M 2016; Epstein et al. 2017); PPI]. Some users expressed a desire for menstrual apps to be location-specific (Hohmann-Marriott, Williams, and Girling 2023).  Using SRT data to inform medical appointments. Most SRTs (particularly menstrual apps) did not make it easy to compile specific data to inform medical appointments [PPI; (Pichon et al. 2021; Epstein et al. 2017)]. Consequently, users and providers felt overwhelmed when trying to use the SRT data to inform care (Pichon et al. 2021).  High health literacy demands. Most SRTs and endometriosis websites provided health information that required high health literacy skills and often lacked plain English summaries (Hirsch et al. 2017; Dinh et al. 2022; Hong et al. 2024).  Online and paper versions. Paper versions of SRTs with printable symptom diaries were described as laborious and more difficult to use when compared to online SRTs (Oikkonen and Temmes 2025). | Digital SRTs personalisation. The ability for digital SRTs to allow personalisation could facilitate their use. SRTs should also consider the complexities of intersecting identities, goals, and diverse user needs to further enhance their usability (Pichon et al. 2021; Hohmann-Marriott 2021).  To increase inclusivity, SRT designs should not assume gender or sexual orientation of users or their partners. The option for logging sex and fertility should optional (Epstein et al. 2017; Pichon et al. 2021). Tools should offer access to multiple languages to facilitate usage across diverse regions and countries (Parker et al. 2022).  SRTs design. SRTs should be designed to accommodate users with irregular cycles or those without menses (Epstein et al. 2017; Pichon et al. 2021; Oikkonen and Temmes 2025). For example, tools should include content informing users that variation and unpredictability across cycles can be normal (PPI). In addition, Endometriosis-specific apps should be able to cater to varying levels of disease.  SRTs functionality. Tools should provide options for users to disable and customise notifications (Pichon et al. 2021; Hong et al. 2024). In addition, apps should also be designed to allow for partial data entry (Hohmann-Marriott, Williams, and Girling 2023).  Transparent and regulated privacy for digital apps. Policies around data confidentiality and privacy should hold tool makers accountable for how they collected, stored, used and shared user’s data (Pichon et al. 2021).  SRTs user agreements and potential implications on use of personal data by tool makers should be informed in an accessible and user-friendly manner – as opposed to arduous and long user agreements (Hohmann-Marriott, Williams, and Girling 2023; Oikkonen and Temmes 2025).  Continuous engagement with SRT. Interventions to enhance SRT engagement should be implemented within the first three months of SRT usage to improve likelihood of retaining user engagement (Elhadad N. 2022). In addition, digital notifications reminding users to track symptoms and functionalities that support self-management might also help ensure sustained engagement (Elhadad N. 2022; McKillop M 2016; Patel et al. 2024; Epstein et al. 2017).  Using SRT data to inform medical appointments. Machine learning and AI can streamline outputs from SRT, making them more user-friendly for both patients and providers to inform care (Pichon et al. 2021). For instance, algorithms could facilitate the recognition of symptom patterns (Pichon et al. 2021) . Additionally, systems should be in place to facilitate electronic sharing of SRT data with GPs, enhancing efficiency in using this information to tailor care (Group 2018). |
| **Factors relating to the organisation of health care, medical understanding of endometriosis and communication** | Differentiation between normal period pain and menstrual disturbances. Providers and patients struggled to differentiate between normal period pain and pain indicating an underlying issue (Armour, Avery, et al. 2022; Hong et al. 2024). This affected SRTs use because users and providers often considered severe period pain as normal, leading to untracked and unrecognised signs of a potential underlying issue.  Enigmatic disease. The heterogeneity of symptoms and poor understanding of the cause of endometriosis contributed to uncertainty regarding its diagnosis and management (Pichon et al. 2021; Oikkonen and Temmes 2025). This made it challenging to determine which symptoms would be meaningful to track for both the patient and provider (Pichon et al. 2021).  Fragmented care. The absence of joined up care combined with limited access to services and different specialities, often resulted in poor patient satisfaction. As a result, patients often taking on a significant administrative responsibility and have to advocate for themselves (Armour, Avery, et al. 2022; Pichon et al. 2021; Group 2018). Despite that, most SRTs lacked the function to aid patients and healthcare providers navigate different health specialities and care pathways (Pichon et al. 2021).  Limited time with GPs. In the UK, the average time of a GP appointment is 9.2 minutes. With such time constraints and a significant amount of symptom data to discuss, it was challenging for both patients and practitioners to have detailed conversations about symptoms, their potential implications, and the direction of care – particularly if the GP lacked clinical knowledge of endometriosis (PPI). | Clinical awareness of endometriosis in primary care. Improved awareness of endometriosis and the provision of resources on the condition can aid GPs in delivering and understanding the role of primary care in women’s health (Dixon et al. 2021). Moreover, there is a need for increased education among GPs regarding SRTs and their use in primary care to ensure effective implementation of SRTs (Frayne et al. 2023; Zhaunova et al. 2023). |
| **Societal factors** | Shame, stigma and taboo surrounding menstruation and symptoms of endometriosis. Many women, girls and AFAB individuals did not feel empowered or comfortable discussing their periods, even though painful menstruation is a key indicator of endometriosis and other underlying issues (Zhaunova et al. 2023). Individuals with endometriosis often expressed feeling invisible or pressured into silence, leading them to hide or downplay their symptoms (Sims et al. 2021). This lack of empowerment and visibility can discourage people from using SRTs. | Insufficient education on menstrual health is leading more young people to seek information from the internet and SRTs. The public relies on apps to access reliable information on menstrual health (Patel et al. 2024; Armour, Avery, et al. 2022; Maslowski et al. 2023; Harper and Botero-Meneses 2022).  College and University-level education were linked with SRT use (Hong et al. 2024).  Childbirth experience significantly impacts symptom tracking, with those who have not experienced childbirth being more likely to track their symptoms. Evidence suggested this is related to a shift in priorities and a lack of time (Hong et al. 2024). |

 Key - AFAB: assigned female at birth; GP: general practitioner; PPI: Patient and Public Involvement.

**Supplementary Table 11** Articles containing the themes used to derive the chains of inference were identified for healthcare professionals and women according to diagnosis status (i.e. whether women were unaware or suspected that their symptoms might be endometriosis, or had a diagnosis of endometriosis)

| **Broader Categories** | **Chains of Influence** | **Derived from the following themes from each study** | **Articles** |
| --- | --- | --- | --- |
| **Patient use of Symptom Reporting Tools independently of medical encounters** | Symptom Reporting Tools can help promote/support patient self-reflection and self-discovery [includes only women’s perspectives] | - Symptom tracking and self-monitoring [diagnosed, undiagnosed] - Awareness of the condition and related factors [diagnosed] - Awareness of endometriosis diagnostic and their symptom patterns improves symptom tracking [diagnosed] - Helps to validate patients' symptoms [diagnosed] | (Pichon et al. 2021; McKillop M 2016; Hohmann-Marriott, Williams, and Girling 2023; Hohmann-Marriott 2021; Levy and Romo-Avilés 2019; Shea 2025; Oikkonen and Temmes 2025; Hong et al. 2024) |
|  | Symptom Reporting Tools can increase the perception that symptoms are worthy of seeking medical help [includes only women’s perspectives] | - Awareness of period pain and menstrual symptoms [Unaware] - Prompts to seek medical help [Unaware/suspected/diagnosed] | (Armour, Avery, et al. 2022; Armour, Parry, et al. 2022; Zhaunova et al. 2023; Hohmann-Marriott, Williams, and Girling 2023; Shea 2025; Oikkonen and Temmes 2025; Hong et al. 2024) |
|  | Symptom Reporting Tools function and functionality – Patients want to track disease lived experience rather than only focus on symptoms [includes only women’s perspectives] | - Symptoms/experiences with Symptom Reporting Tools [diagnosed and suspected] | (Pichon et al. 2021; McKillop M 2016; Scheck et al. 2023; Hohmann-Marriott 2021; Patel et al. 2024; Shea 2025) |
|  | Managing life. Symptom Reporting Tools can help patients understand symptom patterns, triggers and what helps to improve self-management and quality of life OR Increase stress related to tracking [includes only women’s perspectives] | - Symptom tracking and self-monitoring [diagnosed/suspected] - Awareness of the condition and related factors [diagnosed] - Help improve self-management [diagnosed] - Improves quality of life [diagnosed] - Inform self-management [diagnosed] - Validation/empowerment [diagnosed] | (McKillop M 2016; Zhaunova et al. 2023; Pichon et al. 2021; Elhadad N. 2022; Frayne et al. 2023; Hohmann-Marriott, Williams, and Girling 2023; Patel et al. 2024; Broad, Biswakarma, and Harper 2022; Levy and Romo-Avilés 2019; Armour, Avery, et al. 2022; Armour, Parry, et al. 2022; Shea 2025; Oikkonen and Temmes 2025; Hong et al. 2024) |
|  | Managing life/Education aspect. Resources associated with Symptom Reporting Tools can help patients understand symptom patterns, improve menstrual health literacy leading to improved self-management and quality of life [includes only women’s perspectives] | - Awareness of condition and related factors [diagnosed] - Validation/empowerment [diagnosed] - Inform self-management [diagnosed] - Improves quality of life [diagnosed/suspected] | (McKillop M 2016; Pichon et al. 2021; Zhaunova et al. 2023; Hohmann-Marriott, Williams, and Girling 2023; Patel et al. 2024; Armour, Avery, et al. 2022; Armour, Parry, et al. 2022) |
|  | Difficulty of diagnosis creates the need for patients to advocate for themselves [includes only women’s perspectives] | - Symptom tracking and self-monitoring [suspected] - Delay in diagnosis [suspected] - Factor creating barriers (lack of resources, capacity, guidelines) [suspected] | (Pichon et al. 2021; Group 2018) |
| **Symptom Reporting Tool use by women during medical encounter** | Using Symptom Reporting Tools helps patients tag observations and detect patterns which can help to inform appointments OR when ignored by healthcare professionals can increase distress [includes only women’s perspectives] | - Symptom tracking and self-monitoring [diagnosed] - Help understand when and why to seek help [Unaware] - Helps to inform appointments [diagnosed] - Help improve communication [Unaware] - Awareness of condition and related factors [diagnosed] - Lack of validation [diagnosed] | (Pichon et al. 2021; McKillop M 2016; Frayne et al. 2023; Hohmann-Marriott, Williams, and Girling 2023; Scheck et al. 2023; Broad, Biswakarma, and Harper 2022; Armour, Avery, et al. 2022; Armour, Parry, et al. 2022; Shea 2025; Oikkonen and Temmes 2025) |
|  | Using Symptom Reporting Tools to educate providers about patients’ experience with condition and improve communication [includes only women’s perspectives] | - Symptom Reporting Tools can support self-reflection and self-discovery [diagnosed] - Awareness of period pain and menstrual symptoms [Unaware] - Help improve communication between patients and healthcare professionals [diagnosed] - Validation/empowerment [diagnosed] | (Pichon et al. 2021; McKillop M 2016; Frayne et al. 2023; Hohmann-Marriott, Williams, and Girling 2023; Patel et al. 2024; Levy and Romo-Avilés 2019; Armour, Avery, et al. 2022; Armour, Parry, et al. 2022) |
|  | Patients' increased knowledge and self-reflection from Symptom Reporting Tools empower them to be more actively involved during appointments leading to more personalised treatment [includes only women’s perspectives] | - Symptom Reporting Tools can help healthcare professionals prepare for medical encounters [diagnosed, undiagnosed] - Help improve communication between patients and healthcare professionals [diagnosed, undiagnosed] - Awareness of period pain and menstrual symptoms [diagnosed, undiagnosed] | (Pichon et al. 2021; McKillop M 2016; Zhaunova et al. 2023; Hohmann-Marriott, Williams, and Girling 2023; Broad, Biswakarma, and Harper 2022; Armour, Avery, et al. 2022; Armour, Parry, et al. 2022; Shea 2025) |
|  | Validation. Sharing data from Symptom Reporting Tools with healthcare professionals as evidence of symptoms [includes both women’s and healthcare professionals' perspectives] | - Symptom Reporting Tools can improve the relationship between patient and provider [diagnosed, undiagnosed] - Symptom Reporting Tools can help to build trust [diagnosed, undiagnosed] - Awareness of period pain and menstrual symptoms [diagnosed, undiagnosed] | (Pichon et al. 2021; McKillop M 2016; Frayne et al. 2023; Hohmann-Marriott, Williams, and Girling 2023; Broad, Biswakarma, and Harper 2022; Ford, De Togni, and Miller 2021; Oikkonen and Temmes 2025) |
|  | Sharing data from Symptom Reporting Tools with healthcare professionals to inform appointments improved communication and empowered patients to be more actively involved leading to more personalised treatment OR it could deter people from seeking healthcare, or leave patients feeling blamed when treatment/self-management does not work [includes both women’s and healthcare professionals' perspectives] | - Awareness of period pain and menstrual symptoms [diagnosed, undiagnosed] - Symptom Reporting Tools can disturb balance power dynamic [diagnosed, undiagnosed] - Symptom Reporting Tools can help improve patient involvement [diagnosed, undiagnosed] - Symptom Reporting Tools can support treatment and self-management strategies [diagnosed, undiagnosed] - Validation [diagnosed] - Symptom tracking and self-monitoring [diagnosed] | (Pichon et al. 2021; McKillop M 2016; Frayne et al. 2023; Oikkonen and Temmes 2025) |
| **Symptom Reporting Tool use by healthcare professionals during medical encounter** | Healthcare professionals have a different conceptualisation of patient’s health [includes both women’s and healthcare professionals' perspectives] | - Symptom Reporting Tools can help inform further tests and referral [diagnosed, undiagnosed] | (Pichon et al. 2021; Hohmann-Marriott, Williams, and Girling 2023) |
|  | Improve patient-clinician relationship [includes both women’s and healthcare professionals' perspectives] | - Symptom Reporting Tools can help improve healthcare professionals’ knowledge [diagnosed, undiagnosed] - improve patient involvement [diagnosed, undiagnosed] - Validation [diagnosed] - Help improve communication between patients and healthcare professionals [diagnosed, undiagnosed] - more timely diagnosis undiagnosed] - Factor creating barriers (complexity of condition) | (Pichon et al. 2021; Frayne et al. 2023; Shea 2025; Oikkonen and Temmes 2025) |
|  | Improved patient awareness helps to equalise patient-provider relationship and improve the power dynamic OR misinformation can create stress between patient and healthcare professionals [includes both women’s and healthcare professionals' perspectives] | - Symptom Reporting Tools can help improve healthcare professionals’ knowledge [diagnosed, undiagnosed] - Help improve communication between patients and healthcare professionals [diagnosed, undiagnosed] - Better outcomes | (Pichon et al. 2021; McKillop M 2016; Frayne et al. 2023; Patel et al. 2024; Oikkonen and Temmes 2025) |
|  | Symptom Reporting Tools can help inform referral to specialist [includes both women’s and healthcare professionals' perspectives] | - Symptoms Reporting Tools can help improve healthcare professionals’ knowledge [diagnosed, undiagnosed] - Better informed referrals, prompt tests and investigations [diagnosed, undiagnosed] - Better outcomes [diagnosed, undiagnosed] | (Frayne et al. 2023; Armour, Avery, et al. 2022; Armour, Parry, et al. 2022) |
|  | Symptom Reporting Tools improve healthcare professionals’ knowledge of condition/patient and can help inform the next steps regarding care for the patient [includes both women’s and healthcare professionals' perspectives] | - Symptom Reporting Tools help healthcare professionals prepare for medical encounters [diagnosed, undiagnosed] - Help improve communication between patients and healthcare professionals [diagnosed, undiagnosed] - Awareness of period pain and menstrual symptoms [diagnosed, undiagnosed] | (Frayne et al. 2023; Armour, Avery, et al. 2022; Armour, Parry, et al. 2022; Shea 2025) |
|  | Symptom Reporting Tools can help inform initial conversations and steps in care but are not always helpful for determining diagnosis (primary care) [includes both women’s and healthcare professionals' perspectives] | - Improve the relationship between patient and provider [diagnosed, undiagnosed] - Help to build trust [diagnosed, undiagnosed] - Awareness of period pain and menstrual symptoms [diagnosed, undiagnosed] | (Frayne et al. 2023; Hohmann-Marriott, Williams, and Girling 2023; Oikkonen and Temmes 2025) |
|  | Healthcare professionals’ acceptance of Tool and associated outcomes [includes both women’s and healthcare professionals' perspectives] | - Awareness of period pain and menstrual symptoms [diagnosed, undiagnosed] - balance power dynamic [diagnosed, undiagnosed] - Improve patient involvement in their own care and during consultations (patient activation) [diagnosed, undiagnosed] - Support treatment and self-management strategies [diagnosed, undiagnosed] - Validation [diagnosed] - Symptom tracking and self-monitoring [diagnosed] | (Frayne et al. 2023; Hohmann-Marriott, Williams, and Girling 2023; Oikkonen and Temmes 2025) |
